# Supplementary material for: What is known from the existing literature about self-management of pessaries for pelvic organ prolapse? A scoping review
Source: BMJ Open. 2022 Jul 18;12(7):e060223. doi: 10.1136/bmjopen-2021-060223 (PMC9297214; doi:10.1136/bmjopen-2021-060223)
Supplement: Supplementary data [file bmjopen-2021-060223supp002.pdf]

EBSCOhost

Supplementary material 2

Monday, October 18, 2021 4:07:34 PM

| # | Query | Limiters/Expanders | Last Run Via | Results |
|---|---|---|---|---|
| S1 | pessary AND ( self management or self care ) | Expanders - Apply equivalent subjects<br>Search modes - Boolean/Phrase | Interface - EBSCOhost Research Databases<br>Search Screen - Advanced Search<br>Database - CINAHL Plus | 23 |
