# Supplementary material for: What is known from the existing literature about self-management of pessaries for pelvic organ prolapse? A scoping review
Source: BMJ Open. 2022 Jul 18;12(7):e060223. doi: 10.1136/bmjopen-2021-060223 (PMC9297214; doi:10.1136/bmjopen-2021-060223)
Supplement: Supplementary data [file bmjopen-2021-060223supp003.pdf]

Supplementary material 3

Embase <1974 to 2021 May 07>

1	self care/	65562
2	vagina pessary/	3004
3	1 and 2	44
