# Supplementary material for: What is known from the existing literature about self-management of pessaries for pelvic organ prolapse? A scoping review
Source: BMJ Open. 2022 Jul 18;12(7):e060223. doi: 10.1136/bmjopen-2021-060223 (PMC9297214; doi:10.1136/bmjopen-2021-060223)
Supplement: Supplementary data [file bmjopen-2021-060223supp004.pdf]

Supplementary material 4

Ovid MEDLINE(R) <1946 to May Week 1 2021>

1	Pessaries/	1578
2	Self Care/ or Self-Management/	37957
3	1 and 2	16
