# Supplementary material for: What is known from the existing literature about self-management of pessaries for pelvic organ prolapse? A scoping review
Source: BMJ Open. 2022 Jul 18;12(7):e060223. doi: 10.1136/bmjopen-2021-060223 (PMC9297214; doi:10.1136/bmjopen-2021-060223)
Supplement: Supplementary data [file bmjopen-2021-060223supp005.pdf]

Supplementary material 5

APA PsycInfo <1806 to May Week 4 2021>

| | | |
|---|---|---|
| 1 | exp Self-Management/ | 7368 |
| 2 | exp Self-Care/ | 2977 |
| 3 | 1 or 2 | 10087 |
| 4 | pessary.mp. | 9 |
| 5 | pessaries.mp. | 14 |
| 6 | 4 or 5 | 17 |
| 7 | 3 and 6 | 0 |
