# Supplementary material for: What is known from the existing literature about self-management of pessaries for pelvic organ prolapse? A scoping review
Source: BMJ Open. 2022 Jul 18;12(7):e060223. doi: 10.1136/bmjopen-2021-060223 (PMC9297214; doi:10.1136/bmjopen-2021-060223)
Supplement: Supplementary data [file bmjopen-2021-060223supp006.pdf]

Supplementary Material 6-Data Extraction Form

Database	Author and date of publication	Title	Population studied	Context	Methods	Type(s) of pessary	Stage of prolapse	Age of participants	Follow-up	Key findings related to pessary self-management
Medline	Yoshimura et al, 2020	Changes of intravaginal microbiota and inflammation after self-replacement ring pessary therapy compared to continuous ring pessary usage for pelvic organ prolapse.	50 pessary using women	Attending clinic in University hospital in Japan	Laboratory study	Wallace Ring Pessary	Mean 3	Mean 58 years	At 1 month	Reduction in PV discharge with SM, Younger patients tended to learn SM, Abnormal vaginal microbiota as frequently seen in SM group.

Medline	Murray et al, 2016	Vaginal pessaries: can an educational brochure help patients to better understand their care?	60 women with stage 2 or 3 symptomatic prolapse	Attending pessary clinic in hospital Australia	Prospective study with pretest-posttest	Not reported	Not reported	Mean 69 years	At 1 week and 3 months	85% read the SM brochure at 1/52 and 97% at 2/52, Increased satisfaction with SM information given with patient information brochure than with verbally alone, Increased confidence SM with written information, Brochure= increased happiness with information given, increased confidence in SM, increased knowledge re: POP, Increased knowledge re: what a pessary does, increased knowledge re: what to do if a pessary falls out, increased knowledge re: when to use

										oestrogen, increased knowledge regarding what to do if experiencing discharge, vaginal bleeding, a UTI or urinary leakage.
Medline	Tenfelde et al, 2015	Quality of life in women who use pessaries for longer than 12 months.	56 pessary using women	Attending tertiary clinic in USA	Observational cross-sectional study	Not reported	Not reported	Mean 74 years	No follow-up	Most SM women removed pessary daily (40%) or a few times per week (40%). 20% Removed pessary monthly or greater. 44% of women self-managing pessary.
Medline	Manchana, 2011	Ring pessary for all pelvic organ prolapse.	100 women with symptomatic POP	Attending outpatient gynaecology clinic in Thailand	Retrospective case note review	Ring pessary	Mean 3	Median 71 years	Median 13 months	Only 25 women (40%) could SM by themselves or with caregivers assistance. 4 of 25 SM women (16%) and 23 of 37 non SM women (62%) had adverse events e.g. discharge. Self-

										care protocol given as a reason for pessary discontinuation. The ring pessary is easy to teach patient to SM.
Embase	Ma et al, 2021	Vaginal pessary treatment in women with symptomatic pelvic organ prolapse: a long-term prospective study.	312 women with symptomatic prolapse receiving pessary management	Attending tertiary referral centre in China	Prospective observational study	Ring or gell-horn pessary	Mean 3	Mean 69 years	At 5 years	Women were advised to perform weekly self-care. Inability to SM a potential factor in discontinuing pessary management.
Embase	*Chen et al, 2020a	The pessary fits, now what? clinical factors associated with pessary self-management Vs.	218 long term pessary users (excluded those using as interim before surgery or did not complete trial)	Tertiary urogynaecology clinic USA	Retrospective notes review	Not reported	Not reported	Median age 66 years	Not reported	Of 218 women, 159 were self-managing (73%). Increased age, increased BMI, increased prolapse stage, type of pessary and raised POPQ score for Ba, Bp, C and GH increased the

		Clinic management.								odds of office management.
Embase	Holubyeva, 2021	Rates of pessary self-care and the characteristics of patients who perform it.	Notes of 580 women receiving pessary care	Tertiary care centre in USA	Retrospective notes review	Cube, Donut, Gellhorn, Incontinence dish, Incontinence ring, ring, Ring with support and other	Unable to determine due to grouping of prolapse stages	Mean 72 years	No follow-up	31% (174/563) chose to SM. Patients performing SM were seen less frequently (4-6 monthly) whereas non-SM women were seen 1-3 monthly. Less SM experienced PVB (16% vs 26%) and vaginal erosions (10% vs 24%). Patients in SM group significantly younger. Post-menopausal women more likely to want office care. Women with lower stage prolapse (1-2) more likely to SM. SM women more likely to be fitted with a ring

										pessary or ring with support whereas office-based care women more likely to be fitted with a gell-horn or ring with support. Women who opted not to SM had a slightly higher BMI, increased rates of arthritis and cognitive impairment, however none of this was statistically significant.
Embase	*Chen et al, 2020b	Pessary self-managem ent vs. clinic managem ent: Is one more likely to result in surgery?	218 long term pessary users (excluded those using as interim before surgery or did not complete trial)	Tertiary urogyna ecology clinic USA	Retrospec tive notes review	Not reported	Not reported	Median age 66 years	Not reporte d	Of 159 SM women, 30 (19%) had surgery and of 59 office managed women, 20 (35% had surgery.

Embase	Chien et al, 2020	Long-term outcomes of self-management gellhorn pessary for symptomatic pelvic organ prolapse	Notes of 93 women using gell-horn pessary	Tertiary urogynecology unit in Taiwan	Retrospective notes review	Gellhorn pessary	Mean 3	Mean 68 years	Median 50 months	Of 421 women referred for gell-horn SM teaching, 147 elected to proceed-note to self, unsure whether that was just SM or gell-horn itself. Patients taught how to deal with and maintain the gell-horn pessary at home. Of 421 women referred for gell-horn SM teaching, 147 elected to proceed-note to self, unsure whether that was just SM or gell-horn itself. At study end point, 48% had used gell-horn continuously with a mean duration of 50 months. 53 (57%) of women reported AEs. 37

										(40%) had vaginal pain or discomfort with gell-horn in situ or during removal or insertion. Elected for surgery, inconvenience, discomfort while pessary in situ or being removed or inserted or resolution of pessary symptoms. Told to wash and reinsert when bathing.
Embase	*Lammers et al, 2019	Does monthly self-management of vaginal ring pessaries reduce complication rates?	Notes of 75 women taught to SM their pessary	Tertiary referral urogynaecology unit in Australia	Retrospective notes review	PVC ring, continence dish, schaatz introl and gellhorn pessary	Not reported	Median 62 years	Median 23 months	5 women had vaginal erosions (7%) and 3 women had minor complications (4%) which resolved with temporary pessary removal. Overall complication rate was 12%

										which compares with 56% of non SM women (complications classed as PVB or discharge. 5 women had vaginal erosions (7%) and 3 women had minor complications (4%) which resolved with temporary pessary removal. Overall complication rate was 12% which compares with 56% of non SM women (complications classed as PVB or discharge). 4 (7%) proceeded to surgery.
Embase	*Morcuende et al, 2018	Pessary treatment of pelvic organ prolapse: Self care	275 women with POP who had pessary management for	Hospital in America	Retrospective records review	Gellhorn and cube pessaries specifically reported, other types	Not reported	Not reported	Median 17 months	SM women seen yearly or PRN compared with 2-3 monthly. SM group were younger, more

		vs. office-based care.	longer than 3 months			included but no details of type				sexually active, had fewer comorbidities and fewer stage 3 and 4 prolapse. Women in the office care group were more likely to have a space occupying pessary or have required a change in size or type of pessary before. Office care was associated with increased rate of erosions in each group despite adjusting for age, comorbidities, POP stage and pessary type. Non SM women more likely to have vaginal erosions.
Embase	*Hooper et al, 2018	Cube pessary use and quality of	25 women with symptomatic POP	Hospital in America	Prospective, observational	Cube pessary	Not reported	Age range 37-92	Not reported	12 or 25 women were able to self-manage cube pessary.

		life in women with pelvic organ prolapse	fitted with a pessary		multi-site study					Cube pessary improved QOL and vaginal symptoms
Embase	Chan et al, 2019	What are the clinical factors that are predictive of persistent pessary use at 12 months?	152 New pessary fittings in patients	Tertiary care urogyna ecology clinic in Canada	Retrospec tive records review	Ring, schatz, incontinence dish, gellhorn	Not reported	Mean 61 years	12 months	Mode of care: self-care (65% n=98) nurse-care (26% n=39). Protocol advised women not to leave the pessary in situ for longer than 3/12. Many women performed self-removal and cleaning on a daily or weekly basis. Nurse vs Self-care not statistically significant to continuation of pessary use at 12/12.
Embase	*Pizarro-Berdichevs ky et al, 2016	Pessary use in patients with symptom	133 women who have used a pessary for a year or	Hospital in Chile	Prospecti ve questionn aire/note s study	Ring, Gellhorn, Donut and other	81% were stage 3 or 4	Mean 65 years	12 months	68% of patients were able to SM. Inability to SM linked with drop out.

		atic pelvic organ prolapse- 12 month prospective study of factors predictive of subjective improvement	longer POP stage 2+							
Embase	*Ibrahim et al, 2015	Evaluation of pessary service for women with pelvic organ prolapse (POP)	104 women attending pessary clinic	Different hospitals in Scotland	Questionnaire study	Ring or other	Not reported	Not reported	Not reported	9% of women attending the pessary clinic would consider SM. A common reason for not wanting to SM was lack of confidence.
Embase	Khaja and Freeman, 2014	How often should shelf/Gellhorn pessaries be changed? A survey of IUGA	322 Members of IUGA or BSUG	International	Electronic questionnaire study	Not applicable	Not applicable	Not applicable	Not applicable	Self-care usually acceptable with ring pessary. 86% (n=277) do not believe shelf or gell-horn pessaries are suitable for self care.

		urogynae cologists								

Embase	Kearney and Brown, 2014	Self-management of vaginal pessaries for pelvic organ prolapse	134 pessary using women	Tertiary urogynaecology clinic in Cambridge	Quality improvement and evaluation	Ring and sieve pessary	Not reported	29-92 years	6 months	73% of women who agreed to commence SM were SM at 6/12. No difference in ability to SM depending on size of pessary. SM women had increased level of comfort with pessary changes. SM women reported increased convenience. SM women reported increased access to help. SM women reported increased feeling of support. SM women had increased level of comfort with pessary changes. 1 complication of increased discharge advised to remove the pessary less frequently.

										Reasons for pessary removal n=45<br>• 6/12 change (36% n=16)<br>• To clean (31% n=14)<br>• To aid defecation (11% n=5)<br>• Discomfort (n=10 22%)<br>• To have sex (n=6 13%)<br>• Smear or other procedure (n=2 4%)<br>• Holiday (n=1 2%)<br><br>47 women declined SM due to:<br>• Physical barriers (33% and n=15)<br>• Prefers Dr led care (17% n=8)<br>• Clinician found pessary removal/insertion difficult (13% n=6)

										• Nature of intervention (11% n=5)<br>• Too painful (9% n=4)<br>• Emotional barrier (9% n=4)<br>• Feels too old (6% n=3)<br>• Poor cognitive ability (6% n=3)<br>• Travel arrangements to return for teach SM 4% n=2)<br>• Personal circumstance (4% n=2)<br><br>24 women (27%) stopped SM by 6/12 due to:<br>• Manual dexterity (n=9 38%)<br>• PVB (n=1 4%) and therefore Dr led care<br>• Switched to shelf pessary (n=1 4%) and therefore Dr led care

										• Discontinued pessary management (n=11 46%)<br>• Opted for Surgical management (n=2 8%). Frequency of pessary removal and insertion ranged from twice a week to once every 6 months. More women were able/willing to SM with a ring than sieve pessary. 97% of SM women plan to use pessary long term compared with 70% in Dr care group. At 6/12 of SM 17 of 25 women (68%) were no longer considering surgery. Cost saving for Trust and

										commissioners. The teaching package was delivered by a specialist women's health physiotherapist. Recruiting a physiotherapist to this project was not something that we had originally considered as we had initially seen this as a specialist nursing role. However, we believe that by having a physiotherapist (a role which traditionally focuses on rehabilitation and not medical intervention) at the heart of the service, we have been able to develop something which is truly focussed

										on empowering the patient to manage their own condition away from a healthcare setting. During a single 45-minute appointment, informed consent was obtained and the woman was shown and supervised in pessary change. This face to face training was supplemented with written information and an online teaching video. Feedback from the focus group on the information leaflets was used to inform the structure of the teaching video, with increased emphasis placed

										on simple diagrammatic presentation of prolapse and pessary in situ

base	Nemeth et al, 2013.	The cube pessary: An underestimated treatment option for pelvic organ prolapse? Subjective 1-year outcomes.	87 women with stage 2 or 3 prolapse	Urogynaecology clinic in teaching hospital Hungary	Prospective case review	Cube pessary	Median 3	Median 60 years	12 months	85.5% of women rated SM with a cube pessary as very easy or easy
Embase	*Jacobs and Banks, 2010	Pilot study: Pessary use in Bronx women. Would increasing the practice of self-changing pessaries have potential to positively impact	25 women attending a pessary follow-up clinic	Urogynaecology clinic in hospital USA	Questionnaire study	Not reported	Not reported	Mean 78 years	No follow-up	Despite a high level of function in everyday life, only 50% of women felt capable to SM. 1 of 25 women SM. Only a further 2 felt able to attempt. Only 4 would be willing to try. 12 replied that they would like to change their pessary but felt they couldn’t. Body image and function scores

		body image?								equivalent to non pessary users therefore these factors do not appear to link with willingness to SM.
CINAHL	Goh et al, 2020	An audit of vaginal support pessaries for pelvic organ prolapse in western Uganda	93 women with stage 3 or 4 prolapse	Fistula/ prolapse camp in Western Uganda	Audit	R-POP, S-POP and C-POP pessaries	Median 4	Mean 57 years	24 month follow-up	All women (93) taught to SM. 65 trialled pessary for 2 days successfully and were able to SM. Taught to remove once weekly, wash, dry and reinsert. 54 (83%) described SM as easy and had increased confidence in being able to do this after one tutorial.
CINAHL	*Daneel et al, 2016	Does monthly self-removal of vaginal ring pessaries	74 women trained in self-management of ring for POP/SUI	Tertiary urogynaecology clinic in Australia	Audit	Portex ring, Schaatz, Gellhorn, Introl pessary and	Not reported	Median 62 years	Median 23 months	At FU, 66% of women still SM. Complication rate for long term SM women was 10%. Lower complication

		for stress urinary incontinence/ prolapse reduce complication rates? A 5 year audit.				Continence Dish				rate for SM women compared with non-SM women in other studies. Patient wanted surgery, unable to SM due to barriers such as obesity, arthritic fingers) and pessary related complications. Taught to SM by CNS. Women SM rings, Shaatz and gell-horns. Annual FU if SM
CINAHL	Manonai et al, 2018	Vaginal ring pessary use for pelvic organ prolapse: continuation rates and predictors of continued use.	289 women with symptomatic stage 2, 3 and 4 POP	Tertiary centre Thailand	Retrospective chart review	Ring with support	Median 3	Median 71 years	36 months	Self-management protocol included general education re: pessary, self-care coaching re insertion, removal and cleaning, written information and a hotline to call with any emergencies. Self-care of a

										pessary was the only significant factor identified that influenced the continuation rate with pessary management at 3 years. Women who did not require assistance for pessary changes from a healthcare professional or family member were significantly more likely to continue with pessary use.
CINAHL	Storey et al, 2009	Women's experiences with vaginal pessary use.	11 post-menopausal women	Urogynaecology clinic in Canada	Narrative inquiry was used to conduct face-to-face semi-structured interviews.	Not reported	Not reported	All over 65 years	Not applicable	Of 11, only 3 opted to SM. Women referred to being older as a reason not to want to SM. The women who SM reported that they chose it due to confidence in their ability to do so and desire for

										flexibility in when and how they used their pessary. Reasons for not wanting to SM were discomfort ‘touching themselves’, lack of confidence with removal and insertion. Despite this, the women who declined SM showed high levels of confidence in dealing with daily challenges such as adjusting their pessary after opening their bowels. Women described clinic visits as psychological and emotional support as well as physical and pessary care and viewed them as

										social outings. Possible reason not to want to SM.
Handsearch	Meriwether et al, 2015a	The effect of hydroxyquinoline-based gel on pessary associated BV	184 women attending pessary clinic	2 tertiary care centres in USA	Multicentre RCT	Silicone pessary-type not specified	Not reported	Mean 56 years	3 months	Frequency of pessary use or removal did not affect the groups in terms of BV or vaginal symptoms at 2 weeks or 3 months. 34 out of 136 women (25%) who reported frequency of SM, removed at least once a day.
Handsearch	Meriwether et al, 2015b	Sexual function and pessary management among women who are using a pessary for pelvic floor	127 women presenting for a pessary fitting	Two tertiary care centres in USA	Secondary analysis of a randomized trial of new pessary users	Ring pessary, ring with support, ring with knob, incontinence dish, incontinence ring, Shaatz and short-stem Gellhorn	Not reported	Mean 56 years	3 months	Most common reason for r/o pessary, partner could feel during sex (20%) No women reported vaginal discharge or odour as reason for r/o pessary prior to sex. Daily pessary removal association with being sexually

		dysfunctio n								active at 3 months but did not improve sexual function or body image scores. For non-sexually active women, daily pessary removal associated with increased scores on partner related domain.
Handsearc h	Tam et al, 2019	The effect of time interval of vaginal ring pessary replacem ent for pelvic organ prolapse on complicat ion and patient satisfactio n a randomis ed	60 women with stage 1-4 POP who opted for pessary manageme nt	Tertiary urogyne cology center in Hong Kong	Double-blinded, randomiz ed controlled trial	PVC ring pessary	88% stage 1-2	Mean 69 years	6 month	Self care of ring pessary performed by 2 out of 60 women. Many women refused to learn self-care.

		controlled trial								
Handsearch	Fregosi et al, 2018	Changes in the Vaginal Microenvironment as Related to Frequency of Pessary Removal	137 new pessary users attending pessary clinic	2 tertiary care centres in USA	Secondary analysis of RCT	Ring pessary, ring with support, ring with knob, incontinence dish, incontinence ring, Shaatz and short-stem Gellhorn	Not reported	Mean 59 years	3 month	34 (25%) removed the pessary daily, 54 (39%) at least weekly, and 49 (36%) less often than once weekly. Women who removed the pessary less often than weekly were older, using more hormone therapy, and more likely to have BV at baseline. Women who removed their pessaries less often than once weekly had an increased prevalence of anaerobes at 3 months, but no difference in vaginal

										symptoms or pessary satisfaction.
Handsearch	Clemons et al, 2004a	Patient characteristics associated with continued pessary use versus surgery after one year	67 women satisfied with pessary at 2 months for stage 2 or more POP	Urogynaecology clinic in USA	Prospective observational study	Ring and gell-horn	Median stage 3	Mean 72 years	12 months	Ability to change pessary not associated with continued pessary use or decision to opt for surgical management. SM women FU 6-12 monthly, otherwise 2-3 monthly.
Handsearch	Clemons et al, 2004b	Patient satisfaction and changes in prolapse and urinary symptoms in women who were fitted successfully with a pessary for pelvic	73 women with stage 2 or more prolapse successfully fitted with a pessary	Urogynaecology clinic in USA	Prospective observational study	Ring and gell-horn	Median stage 3	Mean 71 years	2 months	21 women (29%) were taught to insert and remove their pessary. All of the women taught to SM used ring pessaries because gell-horn are too difficult to SM.

		organ prolapse								
Handsearch	Kuhn et al, 2008	Sexual organ function in patients with symptomatic prolapse: are pessaries helpful?	73 women with stage 2 or more prolapse	Tertiary referral centre in Switzerland	Prospective observational study	Cube pessary	Not reported	Median 70 years	12 months	(Cube pessary) Patients received advice to remove the pessary as required for sexual intercourse
Handsearch	Bugge et al, 2013	Vaginal pessaries for pelvic organ prolapse and urinary incontinence: a multiprofessional survey of practice	678 members of RCOG, the Association for Continence Advice and the Association of Chartered Physiotherapists in Women's Health in the UK	Members of RCOG the Association for Continence Advice and the Association of Chartered Physiotherapists in Women'	Electronic questionnaire	Not applicable	Not applicable	Not applicable	Not applicable	17.7% (n=84) UK HCP recommend self-care following fitting. 4.1% (n=21) report patient responsible for pessary aftercare at organisation

				s Health in the UK.						
Handsearch	Thys et al, 2020	Effect of pessary cleaning and optimal time interval for follow-up a prospective cohort study	163 women with stage 2 or more primary prolapse fitted with pessary	Outpatient clinic at 3 teaching hospitals. The Netherlands.	Prospective cohort study	Silicone ring with or without support	Median stage 2	Mean 65 years	12 month follow-up	SM performed by 45.2% of women after one year. Patients who performed SM were younger, more frequently diagnosed with atrophy, reported less concomitant SUI and were more often sexually active. No difference between SM and non SM women in vaginal pain, discharge or irritation. No evidence for need to check asymptomatic patients performing SM with a silicone ring. SM more often sexually

										active including vaginal penetration.
Handsearch	Hanson et al, 2006	Vaginal pessaries in managing women with pelvic organ prolapse and urinary incontinence: patient characteristics and factors contributing to success	1216 women referred to nurse led pessary clinic	Nurse led pessary clinic in Canada	Retrospective notes review	Ring, ring with support, Gellhorn, incontinence dish, incontinence ring and the shaatz pessary	Not reported	Median 63 years	3 month follow-up	All patients were encouraged to learn to remove and reinsert the pessary weekly in order to clean the pessary and maintain their independence. Many patients were able to remove and clean their own pessary. Gell-horn and cube pessaries are the most difficult to remove, however a number of women were able to remove their gell-horn.
Handsearch	Abdool et al, 2011	Prospective evaluation of outcome of vaginal pessaries	554 women with symptomatic prolapse treated with either	Specialist urogynaecology unit UK	Prospective questionnaire study	Ring pessary, gellhorn pessary, cube, pessary and donut pessary.	Not reported	Mean 66 years	12 months	Many women preferred not to handle the pessary therefore a ring pessary was inserted for all

		versus surgery in women with symptomatic pelvic organ prolapse	surgery or pessary							sexually active women to avoid this.
Handsearch	Bai et al, 2005	Survey of the characteristics and satisfaction degree of the patients using a pessary	104 women fitted with a pessary and available for FU	Urogynaecology clinic South Korea	Retrospective review of notes and patient completed questionnaire	Ring pessary	Median 3	Mean 70 years	Not reported	The ring pessary is easy to wear and remove and effective. While the donut pessary is hard for a patient to remove by herself because the pessary does not have a notch. 19.1% of women removed their pessary (80% due to it slipping or discomfort)
Handsearch	Clemons et al, 2004c	Risk factors associated with an unsuccessful pessary fitting	100 consecutive women with symptomatic POP fitted with a pessary	Urogynaecology clinic in USA	Prospective observational study	Ring pessary and gell-horn pessary	Median 3	Mean 70 years	2 week	28 women (38%) were taught how to remove, clean and reinsert their pessary (ring or gell-horn pessary).

		trial in women with pelvic organ prolapse								
Handsearch	Sulak et al, 1993	Vaginal pessaries and their use in pelvic relaxation	101 women fitted with a pessary who returned for follow-up	Gynaecology clinic in USA	Retrospective review	Gell-horn pessary used for most participants, other pessary types used not reported	Not reported	Not reported	Not reported	5 of the 50 pessary users (10%) mentioned occasional difficulty with removal of the pessary (gell-horn). Another 5 had to have the pessary removed periodically by a nurse. For gell-horn, all patients (n=96) were advised to remove once or twice a week and leave it out overnight n=35 (70% removed the pessary at least once a week. The remainder removed it at least once every 6 weeks apart

										from one woman who had it in place for 6/12 without issue
Handsearch	*Ramsay et al, 2011	Long-term outcomes of pessary use in women with pelvic organ prolapse	429 women who underwent a pessary trial	Urogynaecology clinic in Hospital in Canada	Retrospective case notes review	Not reported	Not reported	Mean 71 years	Median 35 months	Pessary SM associated with prolonged pessary use. 66% of women were able to SM.
Handsearch	Brown et al, 2016	Defining patient's knowledge and perceptions of vaginal pessaries for prolapse and incontinence	254 women urogynaecology attendees	Urogynaecology clinic at an academic medical centre in the USA	Convenience sample questionnaire study	Not applicable- new patients to urogynaecology services	Not reported	Mean 57 years	No follow-up, data collection amongst new patients to service	Few women indicted concerns about pessary management for example that they would not be able to remove the pessary independently (n=20 16%).

Handsearch	Wu et al, 1997	A simplified protocol for pessary management	110 women receiving pessary management	Clinic in Canada	Prospective modified pessary care protocol followed and long term data collected at follow-up appointments	Ring with support, ring and cube	Unable to determine as reported as combined score for grade of prolapse in each compartment	Mean 65 years	Follow-up data collected up to 54 months	Ring pessary first choice as similar size and shape to diaphragm which patients find easy to insert and remove Most patients chose not to SM their pessary and return for regular office visits for pessary changes
Handsearch	Sarma et al, 2009	Long-term vaginal ring pessary use: discontinuation rates and adverse events	273 women fitted with a ring pessary in a tertiary urogynaecology clinic	Clinic in Australia	Retrospective notes review	Portex ring pessary or Introl bladder neck support device	Not reported	Mean 68 years	Median 7 years	All women who had reasonable manual dexterity were offered the option of self-management. Self-managing women were then seen 12 monthly.
Handsearch	Cundiff et al, 2000	A survey of pessary users by members of the American Urogynae	359 members of AUGS	AUGS members	Questionnaire study	Not applicable	Not applicable	Not applicable	Not applicable	53% (190) doctors reported teaching all patients to change their own pessary. 45% (162) reserve self-

		cology Society								management for a subset of women using support pessaries
