# Supplementary material for: What is known from the existing literature about self-management of pessaries for pelvic organ prolapse? A scoping review
Source: BMJ Open. 2022 Jul 18;12(7):e060223. doi: 10.1136/bmjopen-2021-060223 (PMC9297214; doi:10.1136/bmjopen-2021-060223)
Supplement: Author's manuscript [file bmjopen-2021-060223.draft_revisions.pdf]

BMJ Open is committed to open peer review. As part of this commitment we make the peer review history of every article we publish publicly available.

When an article is published we post the peer reviewers' comments and the authors' responses online. We also post the versions of the paper that were used during peer review. These are the versions that the peer review comments apply to.

The versions of the paper that follow are the versions that were submitted during the peer review process. They are not the versions of record or the final published versions. They should not be cited or distributed as the published version of this manuscript.

BMJ Open is an open access journal and the full, final, typeset and author-corrected version of record of the manuscript is available on our site with no access controls, subscription charges or pay-per-view fees (http://bmjopen.bmj.com).

If you have any questions on BMJ Open's open peer review process please email info.bmjopen@bmj.com

BMJ Open

What is known from the existing literature about self-management of pessaries for pelvic organ prolapse? A scoping review.

Journal:	*BMJ Open*
Manuscript ID	bmjopen-2021-060223
Article Type:	Original research
Date Submitted by the Author:	20-Dec-2021
Complete List of Authors:	Dwyer, Lucy; Manchester University NHS Foundation Trust, The Warrell Unit; The University of Manchester School of Health Sciences, Division of Nursing, Midwifery and Social Work<br>Dowding, Dawn; University of Manchester<br>Kearney, R; Manchester Academic Health Science Centre; University of Manchester
Keywords:	Urogynaecology < UROLOGY, GYNAECOLOGY, STATISTICS & RESEARCH METHODS

SCHOLARONE™
Manuscripts

I, the Submitting Author has the right to grant and does grant on behalf of all authors of the Work (as defined in the below author licence), an exclusive licence and/or a non-exclusive licence for contributions from authors who are: i) UK Crown employees; ii) where BMJ has agreed a CC-BY licence shall apply, and/or iii) in accordance with the terms applicable for US Federal Government officers or employees acting as part of their official duties; on a worldwide, perpetual, irrevocable, royalty-free basis to BMJ Publishing Group Ltd ("BMJ") its licensees and where the relevant Journal is co-owned by BMJ to the co-owners of the Journal, to publish the Work in this journal and any other BMJ products and to exploit all rights, as set out in our licence.

The Submitting Author accepts and understands that any supply made under these terms is made by BMJ to the Submitting Author unless you are acting as an employee on behalf of your employer or a postgraduate student of an affiliated institution which is paying any applicable article publishing charge ("APC") for Open Access articles. Where the Submitting Author wishes to make the Work available on an Open Access basis (and intends to pay the relevant APC), the terms of reuse of such Open Access shall be governed by a Creative Commons licence – details of these licences and which Creative Commons licence will apply to this Work are set out in our licence referred to above.

Other than as permitted in any relevant BMJ Author's Self Archiving Policies, I confirm this Work has not been accepted for publication elsewhere, is not being considered for publication elsewhere and does not duplicate material already published. I confirm all authors consent to publication of this Work and authorise the granting of this licence.

For peer review only

What is known from the existing literature about self-management of pessaries for pelvic organ prolapse? A scoping review.

Dwyer. L[1,2], Dowding. D [2] and Kearney. R[1,3]

Affiliations:

[1] Saint Mary's Hospital, Manchester University NHS Foundation Trust, Manchester Academic Health Science Centre, Hathersage Road, Manchester M13 9WL, UK

[2]Division of Nursing, Midwifery and Social Work, School of Health Sciences, Faculty of Biology, Medicine and Health, University of Manchester, Oxford Road, Manchester, M13 9PL

[3] Institute of Human Development, Faculty of Medical & Human Sciences, University of Manchester, Oxford Road, Manchester, M13 9PL

Corresponding author: Lucy Dwyer

Lucy.dwyer@mft.nhs.uk 0161 276 6910

The Warrell Unit, Saint Mary's Hospital,

Manchester University NHS Foundation Trust

Hathersage Road

Manchester

M13 9WL

Dawn Dowding dawn.dowding@manchester.ac.uk

Rohna Kearney rohna.kearney@mft.nhs.uk

Abstract word count: 271

Main article word Count: 3868

Key words: Pessary, self-management, self-care, prolapse, scoping,

For peer review only - http://bmjopen.bmj.com/site/about/guidelines.xhtml

What is known from the existing literature about self-management of pessaries for pelvic organ prolapse? A scoping review.

ABSTRACT

Objectives

Pelvic organ prolapse (POP) can be managed with a pessary. However, regular follow-up may deter women due to the inconvenience of frequent appointments, as well as preventing autonomous decision making. Pessary self-management, may be a solution to these issues. However, there remains a number of uncertainties regarding pessary self-management. This scoping review aims to map available evidence about pessary self-management to identify knowledge gaps providing the basis for future research.

Design

Scoping review as detailed in the review protocol.

Data sources

A search of MEDLINE, CINAHL, EMBASE and PsycInfo was undertaken to identify relevant articles using the search terms 'pessary' and 'self-management' or 'self-care'.

Data extraction and synthesis

Data relevant to pessary self-management was extracted and the Mixed Methods Appraisal Tool used to assess empirical rigour. Thematic analysis was performed to evaluate the results.

Results

The database search identified 82 publications. After duplicates and articles not meeting the inclusion and exclusion criteria were removed, there were 23 eligible articles. A handsearch revealed a further 19 articles, resulting in a total of 42 publications.

Findings relevant to pessary self-management were extracted and analysed for the emergence of themes. Recurrent themes in the literature were; the characteristics of self-managing women; pessary care; factors associated with decision making about self-management; teaching self-management and cost benefit.

Conclusions

For peer review only - http://bmjopen.bmj.com/site/about/guidelines.xhtml

Pessary self-management may offer benefits to some women without increased risk. Some women do not feel willing or able to self-manage their pessary. However, increased support may help women overcome this. Further in-depth exploration of factors which affect women's willingness to self-manage their pessary is indicated to ensure better understanding and support as available for other conditions.

Ethics and dissemination

No ethical or Health Research Authority (HRA) approval was required prior to undertaking the scoping review. However, it was registered with The Open Science Framework (OSF) (DOI 10.17605/OSF.IO/DNGCP). The findings will inform future research exploring pessary self-management and be disseminated via both a presentation at a national conference and publications in peer reviewed journals.

For peer review only

For peer review only - http://bmjopen.bmj.com/site/about/guidelines.xhtml

ARTICLE SUMMARY

Strengths and limitations of this study

- The scoping review findings provide an overview and synthesis of the current literature about pessary self-management.
- Understanding more about pessary self-management has been highlighted as a research priority by a group of pessary users and healthcare professionals.
- The scoping review was undertaken following systematic and rigorous established guidelines to ensure transparency and reproducibility.
- Critical appraisal revealed recurring methodological limitations within the literature, primarily the use of retrospective case reviews, poor data completeness, failure to account for confounding variables and a lack of representativeness in study populations.

peer review only

For peer review only - http://bmjopen.bmj.com/site/about/guidelines.xhtml

What is known from the existing literature about self-management of pessaries for pelvic organ prolapse? A scoping review.

INTRODUCTION

Mechanical pessaries can effectively reduce symptoms of pelvic organ prolapse and therefore offer an effective alternative to surgery[1] However, the regular pessary follow-up required deters some women from this management option[2-4]. It has been suggested pessary self-management may reduce the frequency of pessary follow-up required. Moreover, it offers women the opportunity to make autonomous decisions about when and how to use their pessary. Nevertheless, there remains a number of uncertainties about pessary self-management including the potential benefits and risks and requirements of self-management teaching and follow-up care[5]. This scoping review aims to map available evidence about pessary self-management to identify knowledge gaps[6] providing the basis for future research.

METHODS

As detailed in the published protocol (Dwyer et al, accepted by BMJ Open-Dec 21), the scoping review was conducted utilising the Joanna Briggs Institute scoping review methodology[7] and reported in accordance with Preferred Reporting Items for Systematic reviews and Meta-Analyses extension for Scoping Reviews PRISMA-ScR guidelines[8]. A search of MEDLINE, CINAHL, EMBASE and PsycInfo was undertaken to identify relevant articles which met the eligibility criteria (Image 1) using the search terms 'pessary' and 'self-management' or 'self-care'.

A hand search of the reference list of non-original research identified during the search but excluded, was conducted for additional publications which met the inclusion and exclusion criteria. Data relevant to the topic of pessary self-management was extracted and critical appraisal of all included publications undertaken using the Mixed Methods Appraisal Tool (MMAT) [9].

PATIENT AND PUBLIC INVOLVEMENT

Members of the public and pessary users have not directly been involved with development of the review protocol or process. However, the need for research exploring pessary self-management was highlighted by The James Lind Alliance (JLA) Priority Setting Partnership for pessary and

For peer review only - http://bmjopen.bmj.com/site/about/guidelines.xhtml

prolapse[10]. Several women with experience of pessaries participated in this partnership either as members of the steering group, by attending the consensus workshop or completing questionnaires. Understanding more about self-management was ranked third out of 20 priorities by the JLA Priority Setting Partnership[10]. The topic of the scoping review has therefore previously been identified and prioritised by patients and members of the public.

RESULTS

The database search identified 82 publications. After duplicates and articles not accessible in the English language were removed, there were 64 remaining publications. After reviewing the identified articles in accordance with the inclusion and exclusion criteria (Image 1), there were 23 eligible articles. A handsearch of reference lists from excluded non-original research papers, revealed a further 19 articles, resulting in a total of 42 publications (Image 2, Supplementary material). Ten of the 42 publications included were solely available as an abstract which prevented thorough understanding and appraisal of both the findings and methodology.

For peer review only

For peer review only - http://bmjopen.bmj.com/site/about/guidelines.xhtml

Table 1-Summary of included studies

**Total number of publications included**	42	
**Continent where research based**		
Africa	1	
Asia	7	
Australasia	4	
Europe	7	
North America	21	
South America	1	
International	1	
**Publication date- Median (Range)**	2015	1993-2021
**Publication type**		
Abstract	10	
Journal article	32	
**Study design**		
Audit	2	
Case note review	14	
Cross sectional	1	
Laboratory study	1	
Observational	17	
Pre-test Post test	1	
Qualitative	1	
Quality improvement evaluation	1	
Randomised controlled trial	2	
Secondary analysis of RCT	2	

For peer review only - http://bmjopen.bmj.com/site/about/guidelines.xhtml

As demonstrated in Table one, the 42 studies included in the review were conducted across all six populated continents. Four (10%) took place in the UK. Half of the eligible studies took place in the USA. Only six (14%) of the eligible studies included in the review were interventional and two of these were secondary analysis of another included publication. A significant majority of the included studies were observational, most being conducted via case note review or questionnaires. A number of included publications report findings from the same sample of women as highlighted in the supplementary material. These findings have been reported separately due to the different aims of each publication.

Critical appraisal of the evidence using the MMAT (Image 3) reveals a number of recurring methodological limitation across the evidence base. Firstly, due to the extent of retrospective case note reviews included, the quality of measurements is unlikely to be good quality due to the lack of a standardised protocol to record details of the pessary fitting and subsequent follow-up[11]. A further limitation is data completeness, due to both high levels of attrition within some of the randomised controlled trials included and a low response rate in many of the quantitative descriptive studies. This may introduce bias to the findings if those who are the most or least satisfied with pessary management are more or less inclined to respond or continue with study participation[11]. Only a small number of the quantitative non-randomised studies included, accounted for confounding variables within their findings by performing stratification, multivariate analysis or matching[11]. In the majority of studies that did not account for confounding variables the validity of the findings is questionable as various factors such as age, hormonal replacement status and prolapse stage could all have influenced the results. One final recurring limitation identified while appraising quantitative descriptive study was the lack of representativeness amongst women participating in the study. Many studies used convenience samples whether only including pessary users who attended follow-up or healthcare professionals who were members of specialist organisations. Therefore, this may limit the generalisability of study findings[11].

Findings relevant to pessary self-management were extracted from the included publications and analysed for the emergence of themes. Recurrent themes in the literature include the characteristics of self-managing women; pessary care; factors associated with decision making about self-management; teaching self-management and cost benefit.

Characteristics of self-managing women

For peer review only - http://bmjopen.bmj.com/site/about/guidelines.xhtml

The extent of pessary self-management is difficult to gauge, though it appears to vary greatly throughout the world. Estimates vary from 18% of UK pessary practitioners to 53% of pessary practitioners in the USA offering pessary self-management[12, 13]. Reasons for this variation are unclear but could be due to differences in care provision possibly due to associated costs of care. It is therefore possible those living in a country with high healthcare costs, may be more willing to self-manage to reduce the costs of attending follow-up appointments.

Several characteristics were associated with women being more likely to self-manage, including being younger[14-21], being pre-menopausal[21], being sexually active[16, 17, 19], having a higher level of education[16], prolonged pessary use[22], having fewer co-morbidities[15-17, 20, 21], a lower stage of prolapse (one or two)[17, 21], a smaller genital hiatus[20] and being diagnosed with atrophy[19].

Age differences between women who self-manage or receive clinician led care may be due to generational attitudes to pessary use or touching one's genitals, or the value associated with perceiving a clinic appointment as a social event with associated psychological and emotional support as proposed by Storey, Aston [14]. Conversely, older women or those with comorbidities may be less likely to self-manage due to a perception of, or actual increased difficulty. However, this has not yet been established.

Space occupying pessaries fill the vaginal cavity and therefore must be removed prior to sexual penetration[23]. Many other pessaries do not need to be removed for penetrative sex[24] However, 70% of women removed their pessary usually or always before sexual activity because their partner could feel the pessary, or due to discomfort[25]. Over half of the women removed the pessary before sexual intercourse after being asked by their partner rather than due to concerns about vaginal discharge or odour[25]. Despite daily pessary removal being associated with sexual activity, this was not correlated with sexual function[25]. Moreover, for women who were not sexually active, frequent pessary removal was associated with an improved relationship with one's partner.

There is disagreement in the literature regarding whether self-management affects the rate of women who choose to continue with pessary management of their prolapse. Self-management has been associated with pessary management continuation[22, 26-28]. Conversely, other studies reported pessary self-management did not affect pessary continuation rates when compared with either nurse or doctor clinician-led care at one year[17, 29, 30]. It is important to note the studies where no relationship was established were based upon shorter-term data. Therefore, it is possible this does not allow sufficient time to measure a difference.

For peer review only - http://bmjopen.bmj.com/site/about/guidelines.xhtml

Pessary care

There is a wide range of pessaries available to manage pelvic organ prolapse and these are typically classed as either a support or space occupying pessary[23]. Frequently used support pessaries in the UK include ring pessaries, rings with support and shaatz pessaries[12]. There is agreement amongst the literature that support pessaries are suitable for women to self-manage, with ring pessaries being 'acceptable'[31] and 'easy'[32-34] to remove and insert. Wu, Farrell [32] suggest women may feel comfortable performing self-management with a ring pessary as it is a similar size and shape to a diaphragm. Whether there is any relationship between willingness or ability to self-manage a pessary, and previous use of a vaginal device has not previously been explored and research into this may provide important insight. Kearney and Brown [2] report more women were willing or able to self-manage with a ring than sieve pessary. Further research to assess whether this is replicated amongst a larger population of women is indicated.

Commonly used space occupying pessaries include shelf, gell-horn and cube pessaries[12, 23]. These work by providing both structural support and filling the vaginal cavity to prevent vaginal descent[23]. Women using space occupying pessaries are less likely to perform self-management than those using support pessaries[17, 21]. Moreover, Clemons, Aguilar [35] states gell-horn pessaries are too difficult to self-manage, a perception shared by many pessary practitioners[13, 31]. Despite this widespread belief, a number of studies suggest self-management of space occupying pessaries such as shelf or gell-horn pessaries is possible[36, 37], if in some cases more difficult[38]. After self-management teaching, 62% and 47% of women continued to self-manage their gell-horn pessary after one and five years, respectively[37]. This compares similarly to the percentages of women self-managing with other types of pessaries. Bai, Yoon [33] state the donut pessary is difficult for a woman to remove independently, however no rigorous evidence to support this statement is provided. More perplexing is Hanson et al's[38] assertion that cube pessaries are amongst the most difficult pessary to remove. Again, there is no rigorous evidence regarding this statement. Furthermore, according to the manufacturers, it is a requirement to self-manage a cube pessary as it should be removed on a nightly basis [39]. This is due to the increased risk of complications due to the greater surface area covered by the cube pessary and the mild suction[40].

At present there is a lack of evidence about the required frequency of pessary removal by self-managing women to inform practice. The frequency of pessary removal and insertion will vary in accordance with the reasons for self-management. The most frequently reported frequency of pessary removal and insertion was daily to a couple of times a week[15-17, 30]. A number of

For peer review only - http://bmjopen.bmj.com/site/about/guidelines.xhtml

protocols provided specific advice regarding frequency of pessary removal. Whether removal each time they bathed[37] or removal and to leave the pessary out overnight once or twice a week[28, 41]. Seventy percent (n=35) of women followed this advice given and removed the pessary at least weekly[41]. One self-managing woman left the gell-horn pessary in situ for six months without removal and experienced no issues. This is unsurprising, as for most UK centres, standard clinician-led care is to follow-up pessaries on a six-monthly basis[42]. On this basis, Kearney and Brown [2] recommended self-managing women remove their pessary at a minimum of six monthly. Chan, Hyakutake [30] advised women not to leave the pessary in situ for longer than 3 months, which mirrored clinician-led care at that organisation. A self-management protocol advising women to remove their pessary on a weekly basis was cited as the reason for pessary discontinuation as women found it too burdensome[34]. While there is no evidence to inform guidance about recommended frequency of pessary removal and insertion, instructing women to remove their pessary very frequently may deter women from self-management but also reduce the autonomy self-management offers.

The majority of studies suggest pessary self-management offers benefits in terms of women's comfort, convenience, perceived access to help and support and feeling of independence[2, 38]. Self-management reduces complications when compared with clinician-led care, with an overall complication rate for self-managing women of 10-16%[34, 36, 43], compared to complication rates of 62% for women receiving clinician-led care[34]. Studies report a lower rate of vaginal erosions for self-managing women[17, 21]. However, no difference was reported between women self-managing or receiving clinician led care regarding vaginal pain, discharge or irritation[19]. Vaginal pain or discomfort was experienced by 40% (n=37) of women during removal or insertion of their gell-horn pessary[37]. As there was no comparison with women receiving clinician led care it is not known whether the pain and discomfort reported by women is comparable to that experienced by women during clinician-led pessary changes.

There is mixed evidence for the effect on vaginal discharge, with one study reporting an increase for one woman who commenced self-management[2] and another an increase in discharge for women who left their pessary in situ continuously[18]. Despite identifiable differences in the vaginal microenvironment associated with frequency of removal, this did not appear to be clinically significant with regards to women's symptoms after three months[44]. It is possible if conducted over a longer period, such as six months which is more akin to the typical length of time a pessary is left in situ[42], greater differences might have been identified. Furthermore, due to the frequency of removal the women were grouped into, the findings cannot be generalised to the large population of women who do not remove their pessary at all.

For peer review only - http://bmjopen.bmj.com/site/about/guidelines.xhtml

Factors associated with decision making about self-management

Many women refused to self-manage their pessary when offered this option, preferring to return regularly for clinician-led care instead[32, 45]. The percentage of women willing to self-manage their pessary varies significantly, from 3%-83%[14, 20, 21, 30, 35, 45-48]. Reports of women's levels of ability to self-manage their pessary also differs extensively between different studies, ranging between 40-86%[2, 19, 22, 26, 34, 36, 40, 43, 49]. Furthermore, ability to self-manage a pessary does not appear to be a straightforward concept. Twenty percent of women reported occasional difficulty removing their pessary or required assistance from a healthcare professional[41]. After teaching, 17% of women initially found self-management difficult, however, this reduced significantly with repeated attempts[50]. It is unclear why self-management ability may fluctuate. Manual dexterity was cited as an important aspect of self-management ability[51]. Kearney and Brown [2] found issues with this to be the most common barrier, causing seven percent of women to discontinue self-management after six months.

Three studies explored the reasons why women self-managed their pessary. Mostly women removed or readjusted their pessary when it was slipping or uncomfortable[33], or because they desired flexibility in how and when they used their pessary[14]. After six months of self-management the most frequently cited reason for pessary removal was removal and reinsertion at six months as advised by their clinician (36%, n=16). This was followed by removal for cleaning (31%, n=14), removal to aid defecation (11%, n=5), removal due to discomfort (22%, n=10), removal to have sex (13%, n=6) removal for a smear or other procedure (4%, n=2) and removal before a holiday (2%, n=1). It is possible the reasons for removal may be different if collected over a longer period than six months. However, it offers clinicians some understanding into self-management behaviours of women.

Women who experienced pessary related complications were also less likely to opt for self-management, or to discontinue self-management whether due to patient request[36] experiencing unexpected bleeding, or changing to a pessary not suitable for self-management[2]. Physical barriers were the most cited reason women gave to Kearney and Brown [2] for non-participation in the study. However, further detail was not provided therefore it is unclear exactly what these physical barriers were. Another physical barrier to self-management reported by 6% of women was feeling 'too old'. Whether age as a barrier relates to physical ability, a mind-set, or a combination of the two is not clear.

For peer review only - http://bmjopen.bmj.com/site/about/guidelines.xhtml

A reason many women gave for not wanting to self-manage was a lack of confidence[14, 52]. However, despite a lack of confidence undertaking the self-management role, the same women demonstrated high levels of confidence addressing daily challenges with their pessary, such as readjusting it as required[14]. It may also be women feel more able to access support if receiving clinician led care. Conversely, women who commenced pessary self-management reported better access support than those receiving clinician led care[2]. The intimate nature of pessary care is also a consideration, some women reported feeling uncomfortable touching their genitalia[14]. This was also identified by[2] where 11% of women declined self-management due to the nature of pessary care. A similar issue reported by Abdool, Thakar [53] was many women expressing a preference not to handle a pessary. Thirteen percent of women declined self-management, reporting clinicians found removal or insertion difficult and therefore they did not feel able to self-manage the pessary independently[2].

Teaching self-management

At present there are no evidence-based guidelines to specify the requirements of self-management teaching[54]. The identified literature provides some evidence regarding the included constituents of self-management teaching. Where reported, self-management teaching took between 45 minutes and an hour[2, 55]. The level of detail provided about self-management teaching varied greatly. Therefore, it was not always clear whether details of basic instructions were simply a brief description, or an accurate representation of the teaching provided. In all identified studies which provided details about the teaching, women were given instructions on removal and insertion of the pessary[2, 27, 50, 55]. Kearney and Brown [2] specified that women were supervised practicing a pessary change however this was not mentioned elsewhere.

Kearney and Brown [2] state that as well as face to face training, women were provided with written information and access to an online video. Both were designed utilising feedback from pessary users who attended a focus group. Women who received written information were more satisfied, more confident and demonstrated increased knowledge regarding every aspect of pessary care covered by the brochure when compared with women solely given verbal information[48].

Within the identified literature women were taught to self-manage by specialist nurses[36, 55] or specialist women's health physiotherapist[2]. Kearney and Brown [2] originally intended to have a specialist nurse undertake this role. However, they believe having a physiotherapist who would typically perform a rehabilitative role rather than delivering medical interventions was beneficial to

For peer review only - http://bmjopen.bmj.com/site/about/guidelines.xhtml

the service evaluation. Only one study evaluated outcomes following self-management teaching. [50] found after only one tutorial, new pessary using women had increased confidence to self-manage their pessary and described it as 'easy'.

Cost benefit

While the primary goal of pessary self-management is an improved experience for women, there is also the potential for cost savings if the number of follow-up appointments required can be reduced both for the woman and the service[27, 50]. While the cost of attending frequent follow-up may be a consideration for women attending appointments anywhere, it is important to clarify the women audited by Goh, Thayalan [50] lived in a 'poor' rural farming area of Uganda, one of the poorest countries in the world[56]. Therefore, the impact of costs to travel to an appointment are likely to be more of a consideration, or deterrent, for women in this audit compared with the general population. Detailed cost analysis of self-management versus clinician-led care suggests potential for annual cost savings of £5,500 for the Trust and £8,400 for commissioners for 50 women[2]. This calculation is based upon 90 minutes of a band six nurse or physiotherapist's time to provide initial self-management teaching and subsequent telephone follow-up. It is important to note, the significant cost savings calculated are based upon clinician-led pessary care being provided by doctors, whereas at most organisations in the UK it is provided by a combination of doctors and nurses[57]. This may affect projected cost savings.

DISCUSSION

Despite a number of factors being associated with likelihood to self-manage[14-22], a causative relationship has not been established with any. As physical ability does not appear to affect willingness to self-manage a pessary[41, 50], further exploration is required to enable us to understand why such a large number of women are unwilling to undertake this role[14, 20, 21, 30, 32, 35, 45-48].

As suggested in the literature, it is unsurprising with increasing practice and confidence, self-management would get easier[50]. It may also be due to pessary related factors such as the position of the pessary changing and becoming harder to reach or more immovable. Alternatively, it might be that dynamic characteristics of the woman such as hormonal changes affecting the flexibility or lack of, of the surrounding vaginal tissues, physical flexibility and dexterity fluctuations cause occasional difficulty. Due to the fluctuating nature of self-management ability[41], it is questionable whether a decision can be made upon one assessment. Despite this, there is no robust evidence regarding the

For peer review only - http://bmjopen.bmj.com/site/about/guidelines.xhtml

skills and ability required to self-manage a pessary, therefore further research in this area is indicated.

The finding of the scoping review demonstrate the lack of evidence to guide decision making regarding pessary self-management. However, there is potential for the weight of evidence to appear greater than it is due to repetition. Ten of the included studies had been solely published in abstract format despite many of them being presented a number of years ago[17, 20, 22, 26, 36, 43, 47, 49, 52, 58]. The decision was made to include these abstracts despite not being published in full to ensure very recent data was included. However, it has highlighted the potential for publication bias in the literature related to self-management.

Despite critical appraisal of evidence not being a traditional component of scoping reviews[7], assessment of the quality of the included papers has enabled us to identify recurring themes in the methodological limitations of the evidence base. This highlights the need for well-designed research studies, particularly randomised controlled trials within this field to improve rigour within the literature. A further limitation is restriction to the four databases searched which may result in failure to identify eligible, relevant publications. The search terms utilised included 'self-management' and 'self-care'. However, it is possible not all centres use these terms to describe teaching women to remove and insert their pessaries. In which case only instances where authors use the terms self-management or self-care or perceive self-management of pessaries as something other than standard care will have been identified.

In conclusion, despite the lack of rigorous evidence, it appears pessary self-management offers benefits to some women[2, 38] with no increased risk of complications[17, 21, 34, 36, 43]. It is clear some women do not feel willing or able to self-manage their pessary[2, 14, 19-22, 26, 30, 32, 34-36, 40, 43, 45-49]. However, it is uncertain whether increased support to help women overcome these issues or concerns may address this. Further research is indicated to clarify the follow-up required for self-managing women, as well as the constituents of effective self-management teaching. The findings of the TOPSY study[54] expected to be published in 2022 are anticipated to clarify certain aspects of self-management care. However, further in-depth exploration of factors which affect women's willingness to self-manage their pessary is indicated to ensure the issue is better understood and women are better supported to self-manage their prolapse as those with other conditions have been previously.

For peer review only - http://bmjopen.bmj.com/site/about/guidelines.xhtml

AUTHORS CONTRIBUTIONS

Lucy Dwyer devised the scoping review question, methodology, performed the literature search, data extraction and drafted this manuscript.

Rohna Kearney substantively contributed to the development of the scoping review question, methodology, performed data extraction and critical appraisal of a subsample of the included studies and has revised and approved this manuscript.

Dawn Dowding substantively contributed to the development of the scoping review question, methodology and revised and approved this manuscript.

COMPETING INTERESTS STATEMENT

Lucy Dwyer and Rohna Kearney are co-applicants of the NIHR/HTA funded Treatment of Prolapse with Self-Care Pessary (TOPSY) study.

FUNDING

Lucy Dwyer, Clinical Doctoral Research Fellow, NIHR300519 is funded by Health Education England (HEE) / National Institute for Health Research (NIHR) for this research project. The views expressed in this publication are those of the author(s) and not necessarily those of the NIHR, NHS or the UK Department of Health and Social Care.

DATA SHARING STATEMENT

No additional data available.

For peer review only

For peer review only - http://bmjopen.bmj.com/site/about/guidelines.xhtml

LIST OF TABLES, IMAGES AND SUPPLEMENTARY MATERIAL

Table 1 Summary of studies

Image 1-Inclusion criteria

Image 2-Review flow chart

Image 3- MMAT results

Supplementary material-Table of studies

Supplementary material-CINAHL search

Supplementary material-Embase search

Supplementary material-Medline search

Supplementary material-PsycINFO search

Supplementary material-PRISMA checklist

For peer review only

REFERENCES

1. Lone, F., R. Thakar, and A.H. Sultan, *One-year prospective comparison of vaginal pessaries and surgery for pelvic organ prolapse using the validated ICIQ-VS and ICIQ-UI (SF) questionnaires.* International Urogynecology Journal, 2015. **26**(9).
2. Kearney, R. and C. Brown, *Self-management of vaginal pessaries for pelvic organ prolapse.* BMJ Quality Improvement Reports, 2014. **3**(1).
3. Basu, M., B. Wise, and J. Duckett, *A qualitative study of women's preferences for treatment of pelvic floor disorders.* BJOG: An International Journal of Obstetrics & Gynaecology, 2011. **118**(3): p. 338-344.
4. Nguyen, J.N. and C.R. Jones, *Pessary treatment of pelvic relaxation: Factors affecting successful fitting and continued use.* Journal of Wound, Ostomy and Continence Nursing, 2005. **32**(4).
5. Bugge, C., et al., *Does self-management of vaginal pessaries improve care for women with pelvic organ prolapse?*, in *The BMJ*. 2021.
6. Munn, Z., et al., *Systematic review or scoping review? Guidance for authors when choosing between a systematic or scoping review approach.* BMC Medical Research Methodology, 2018. **18**(1).
7. Peters, M., et al., *Chapter 11: Scoping Reviews (2020 version).* JBI Reviewer's Manual, 2020.
8. Tricco, A.C., et al., *PRISMA extension for scoping reviews (PRISMA-ScR): Checklist and explanation*, in *Annals of Internal Medicine*. 2018.
9. Hong, Q.N., et al., *The Mixed Methods Appraisal Tool (MMAT) version 2018 for information professionals and researchers.* Education for Information, 2018. **34**(4): p. 285-291.
10. Lough, K., et al., *Shared research priorities for pessary use in women with prolapse: Results from a James Lind Alliance Priority Setting Partnership.* BMJ Open, 2018. **8**(4).
11. Sterne, J., J. Higgins, and B. Reeves, *on behalf of the development group for ACROBAT-NRSI, 2014. A Cochrane Risk Of Bias Assessment Tool: for Non-Randomized Studies of Interventions (ACROBAT-NRSI), Version 1.0. 0*. 2014.
12. Bugge, C., S. Hagen, and R. Thakar, *Vaginal pessaries for pelvic organ prolapse and urinary incontinence: A multiprofessional survey of practice.* International Urogynecology Journal and Pelvic Floor Dysfunction, 2013. **24**(6).
13. Cundiff, G., *A survey of pessary use by members of the american urogynecologic society.* Obstetrics & Gynecology, 2000. **95**(6).
14. Storey, S., et al., *Women's experiences with vaginal pessary use.* Journal of Advanced Nursing, 2009. **65**(11).
15. Tenfelde, S., et al., *Quality of life in women who use pessaries for longer than 12 months.* Female Pelvic Medicine and Reconstructive Surgery, 2015. **21**(3).
16. Meriwether, K.V., et al., *The effect of hydroxyquinoline-based gel on pessary-associated bacterial vaginosis: A multicenter randomized controlled trial.* American Journal of Obstetrics and Gynecology, 2015. **213**(5).
17. Morcuende, I., et al., *Pessary Treatment of Pelvic Organ Prolapse: Self Care vs. Office-based Care.* PFD Week 2018, 2018.
18. Yoshimura, K., et al., *Changes of intravaginal microbiota and inflammation after self-replacement ring pessary therapy compared to continuous ring pessary usage for pelvic organ prolapse.* Journal of Obstetrics and Gynaecology Research, 2020. **46**(6).
19. Thys, S.D., et al., *Effect of pessary cleaning and optimal time interval for follow-up: a prospective cohort study.* International Urogynecology Journal, 2020. **31**(8).
20. Chen, Y.B., et al., *The pessary fits, now what? clinical factors associated with pessary self-management Vs. Clinic management.* Female Pelvic Medicine and Reconstructive Surgery, 2020. **26**(10 SUPPL 1): p. S132-S133.

For peer review only - http://bmjopen.bmj.com/site/about/guidelines.xhtml

21. Holubyeva, A., et al., *Rates of Pessary Self-Care and the Characteristics of Patients Who Perform it.* Female pelvic medicine & reconstructive surgery, 2021. **27**(3).
22. Ramsay, S., F. Bouchard, and L.M. Tu, *Long term outcomes of pessary use in women with pelvic organ prolapse.* Neurourology and Urodynamics, 2011. **30**(6).
23. Dwyer, L. and R. Kearney, *Conservative management of pelvic organ prolapse.* Obstetrics, Gynaecology & Reproductive Medicine, 2018. **28**(1): p. 15-21.
24. Rantell, A., *Vaginal Pessaries for Pelvic Organ Prolapse and Their Impact on Sexual Function.* Sex Med Rev, 2019. **7**(4): p. 597-603.
25. Meriwether, K.V., et al., *Sexual Function and Pessary Management among Women Using a Pessary for Pelvic Floor Disorders.* The Journal of Sexual Medicine, 2015. **12**(12).
26. Pizarro-Berdichevsky, J., et al., *Pessary use in patients with symptomatic pelvic organ prolapse-12 month prospective study of factors predictive of subjective improvement.* International Urogynecology Journal and Pelvic Floor Dysfunction, 2016. **27**(1).
27. Manonai, J., S. Sarit-Apirak, and U. Udomsubpayakul, *Vaginal ring pessary use for pelvic organ prolapse: Continuation rates and predictors of continued use.* Menopause, 2019. **26**(6).
28. Ma, C., et al., *Vaginal pessary treatment in women with symptomatic pelvic organ prolapse: a long-term prospective study.* Menopause (New York, N.Y.), 2021. **28**(5).
29. Clemons, J.L., et al., *Patient characteristics that are associated with continued pessary use versus surgery after 1 year.* American Journal of Obstetrics and Gynecology, 2004. **191**(1).
30. Chan, M.C., et al., *What Are the Clinical Factors That Are Predictive of Persistent Pessary Use at 12 Months?* Journal of Obstetrics and Gynaecology Canada, 2019. **41**(9).
31. Khaja, A. and R.M. Freeman, *How often should shelf/Gellhorn pessaries be changed? A survey of IUGA urogynaecologists.* International Urogynecology Journal, 2014. **25**(7).
32. Wu, V., et al., *A simplified protocol for pessary management.* Obstetrics and Gynecology, 1997. **90**(6).
33. Bai, S.W., et al., *Survey of the characteristics and satisfaction degree of the patients using a pessary.* International Urogynecology Journal, 2005. **16**(3).
34. Manchana, T., *Ring pessary for all pelvic organ prolapse.* Archives of Gynecology and Obstetrics, 2011. **284**(2).
35. Clemons, J.L., et al., *Patient satisfaction and changes in prolapse and urinary symptoms in women who were fitted successfully with a pessary for pelvic organ prolapse.* American Journal of Obstetrics and Gynecology, 2004. **190**(4).
36. Daneel, L., N.I. te West, and K.H. Moore, *Does monthly self-removal of vaginal ring pessaries for stress incontinence/ prolapse reduce complication rates? A 5 year audit.* Neurourology and Urodynamics, 2016.
37. Chien, C.W., et al., *Long-term outcomes of self-management gellhorn pessary for symptomatic pelvic organ prolapse.* Female Pelvic Medicine and Reconstructive Surgery, 2020. **26**(11).
38. Hanson, L.A.M., et al., *Vaginal pessaries in managing women with pelvic organ prolapse and urinary incontinence: Patient characteristics and factors contributing to success.* International Urogynecology Journal, 2006. **17**(2).
39. Cooper-Surgical. *Cube and Tandem Cube Pessary Instructions for Use.* 2017; Available from: www.coopersurgical.com/product-resources/7c3c5e98-68d5-4d44-b2fc-3d6bae7d2fad_Cube-and-Tandem-Cube-Pessary-Instructions-for-Use.pdf
40. Nemeth, Z., S. Nagy, and J. Ott, *The cube pessary: An underestimated treatment option for pelvic organ prolapse? Subjective 1-year outcomes.* International Urogynecology Journal and Pelvic Floor Dysfunction, 2013. **24**(10).
41. Sulak, P.J., T.J. Kuehl, and B.L. Shull, *Vaginal pessaries and their use in pelvic relaxation.* Journal of Reproductive Medicine for the Obstetrician and Gynecologist, 1993. **38**(12).

For peer review only - http://bmjopen.bmj.com/site/about/guidelines.xhtml

42. Gorti, M., G. Hudelist, and A. Simons, *Evaluation of vaginal pessary management: A UK-based survey.* Journal of Obstetrics and Gynaecology, 2009. **29**(2).
43. Lammers, K., et al., *Does monthly self-management of vaginal ring pessaries reduce complication rates?* International Urogynecology Journal, 2019. **30**(1 Supplement): p. S271-S272.
44. Fregosi, N.J., et al. *Changes in the Vaginal Microenvironment as Related to Frequency of Pessary Removal*. in *Female Pelvic Medicine and Reconstructive Surgery*. 2018.
45. Tam, M.S., et al., *The effect of time interval of vaginal ring pessary replacement for pelvic organ prolapse on complications and patient satisfaction: A randomised controlled trial.* Maturitas, 2019. **128**.
46. Clemons, J.L., et al., *Risk factors associated with an unsuccessful pessary fitting trial in women with pelvic organ prolapse.* American Journal of Obstetrics and Gynecology, 2004. **190**(2).
47. Jacobs, S. and E. Banks, *Pilot study: Pessary use in bronx women. Would increasing the practice of self-changing pessaries have potential to positively impact body image?* Journal of Pelvic Medicine and Surgery, 2010. **16**(5 SUPPL. 2): p. S143-S144.
48. Murray, C., E. Thomas, and W. Pollock, *Vaginal pessaries: can an educational brochure help patients to better understand their care?* Journal of Clinical Nursing, 2017. **26**(1-2).
49. Hooper, G., et al., *Cube pessary use and quality of life in women with pelvic organ prolapse.* Female Pelvic Medicine and Reconstructive Surgery, 2018. **24**(5).
50. Goh, J.T.W., K. Thayalan, and H.G. Krause, *An audit of vaginal support pessaries for pelvic organ prolapse in western Uganda.* Australian and New Zealand Continence Journal, 2020. **26**(2).
51. Sarma, S., T. Ying, and K.H. Moore, *Long-term vaginal ring pessary use: Discontinuation rates and adverse events.* BJOG: An International Journal of Obstetrics and Gynaecology, 2009. **116**(13).
52. Ibrahim, S., et al., *Evaluation of pessary service for women with pelvic organ prolapse (POP).* International Journal of Gynecology and Obstetrics, 2015. **131**(SUPPL. 5): p. E164.
53. Abdool, Z., et al., *Prospective evaluation of outcome of vaginal pessaries versus surgery in women with symptomatic pelvic organ prolapse.* International Urogynecology Journal, 2011. **22**(3).
54. Hagen, S., et al., *Clinical and cost-effectiveness of vaginal pessary self-management compared to clinic-based care for pelvic organ prolapse: protocol for the TOPSY randomised controlled trial.* Trials, 2020. **21**(1): p. 1-13.
55. Kuhn, A., et al., *Sexual and organ function in patients with symptomatic prolapse: are pessaries helpful?* Fertility and Sterility, 2009. **91**(5).
56. The World Bank. *The Uganda Poverty Assessment Report 2016*. 2016; Available from: https://thedocs.worldbank.org/en/doc/381951474255092375-0010022016/original/UgandaPovertyAssessmentReport2016.pdf.
57. Dwyer, L., E. Stewart, and A. Rajai, *A service evaluation to determine where and who delivers pessary care in the UK.* Int Urogynecol J, 2021. **32**(4): p. 1001-1006.
58. Chen, Y.B., et al., *Pessary self-management vs. clinic management: Is one more likely to result in surgery?* Female Pelvic Medicine and Reconstructive Surgery, 2020. **26**(10 SUPPL 1): p. S151-S152.

For peer review only - http://bmjopen.bmj.com/site/about/guidelines.xhtml

Image 1-Inclusion and Exclusion criteria

Inclusion criteria

- Original research
- Pessary for POP
- Published in English language
- Focuses on self-management of pessary for POP

Exclusion criteria

- Not relevant to subject area
- Not published in English language
- Not original research including case reports

For peer review only

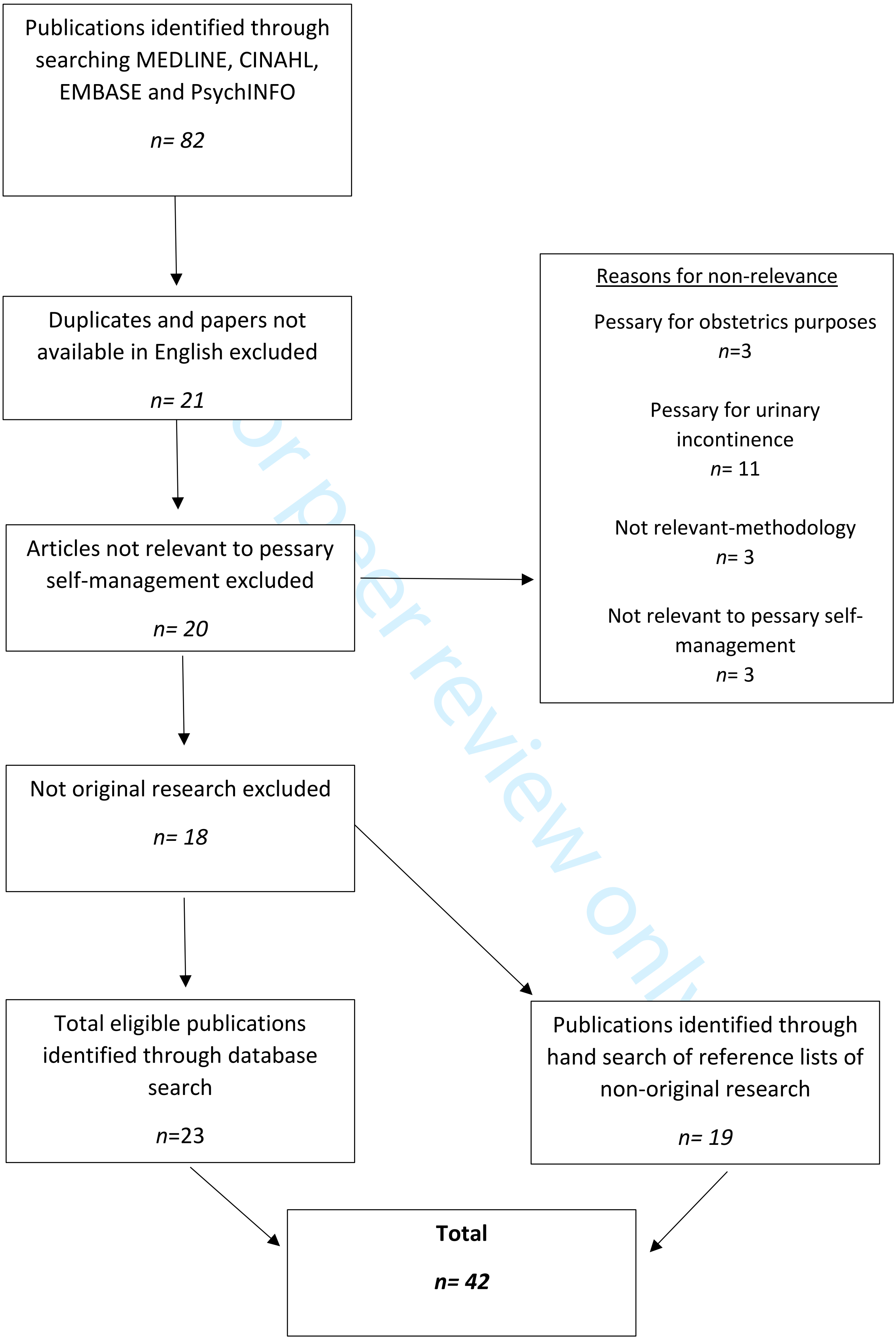
Publications identified through searching MEDLINE, CINAHL, EMBASE and PsychINFO
n= 82
Duplicates and papers not available in English excluded
n= 21
Articles not relevant to pessary self-management excluded
n= 20
Reasons for non-relevance
Pessary for obstetrics purposes
n=3
Pessary for urinary incontinence
n= 11
Not relevant-methodology
n= 3
Not relevant to pessary self-management
n= 3
Not original research excluded
n= 18
Total eligible publications identified through database search
n=23
Publications identified through hand search of reference lists of non-original research
n= 19
Total
n= 42

Image Two-MMAT

Studies	Criteria from the Mixed Methods Appraisal Tool																								
	1.1	1.2	1.3	1.4	1.5	2.1	2.2	2.3	2.4	2.5	3.1	3.2	3.3	3.4	3.5	4.1	4.2	4.3	4.4	4.5	5.1	5.2	5.3	5.4	5.5
Abdool et al, 2011											1	1	1	1	1										
Bai et al, 2005																1	0	0	1	1					
Brown et al, 2016																1	0	1	0	1					
Bugge et al, 2013																1	0	1	0	1					
Chan et al, 2019											1	1	0	1	1										
*Chen et al, 2020a and Chen et al, 2020b**											1	0	1	1	1										
Chien et al, 2020											0	0	0	0	1										
Clemons et al. 2004a, Clemons et al. 2004b and Clemons et al. 2004c**											1	1	1	1	1										
Cundiff et al, 2000																1	1	1	0	1					
*Daneel et al, 2016																1	1	0	1	0					
Goh et al, 2020																1	0	1	1	1					
Hanson et al, 2006											1	0	1	0	1										
Holubyeva et al, 2021											0	0	1	0	1										
*Hooper et al, 2018											1	1	1	0	1										
*Ibrahim et al, 2015																1	1	1	0	1					
*Jacobs and Banks, 2010																1	1	1	0	1					
Kearney and Brown, 2014											1	0	1	0	1										
Khaja and Freeman, 2014																1	0	1	0	1					
*Lammers et al, 2019											1	0	1	0	1										
Kuhn et al, 2008																1	1	1	1	1					
Ma et al, 2001											1	1	1	1	1										
Manchana, 2011											1	1	1	0	1										
Manonai et al, 2018											1	0	0	0	1										

For peer review only

Meriwether et al, 2015a, Meriwether et al, 2015b and Fregosi et al, 2018**						1	1	0	1	1															
*Morcuende et al, 2018											1	0	1	0	0										
Murray et al, 2017											1	1	1	0	1										
Nemeth et al, 2013																1	1	1	1	1					
*Pizarro-Berdichevsky et al, 2016											1	1	1	1	1										
Ramsay et al, 2011											1	0	0	1	1										
Sarma et al, 2009																1	1	0	0	1					
Storey et al, 2009	1	1	1	1	1																				
Sulak et al, 1993																0	0	0	1	1					
Tam et al, 2019						1	1	1	1	1															
Thys et al, 2020											1	1	0	0	1										
Tenfelde et al, 2015																1	1	1	1	1					
Wu et al, 1997																1	1	1	1	1					
Yoshimura et al, 2020											1	1	1	0	0										

* Abstract

** Clustered studies where secondary analysis of the same sample of women was performed

For peer review only

http://mixedmethodsappraisaltoolpublic.pbworks.com/w/file/fetch/140056890/Reporting%20the%20results%20of%20the%20MMAT.pdf

Supplementary Material-Data Extraction Form

Database	Author and date of publication	Title	Population studied	Context	Methods	Key findings related to pessary self-management
Medline	Yoshimura et al, 2020	Changes of intravaginal microbiota and inflammation after self-replacement ring pessary therapy compared to continuous ring pessary usage for pelvic organ prolapse.	50 pessary using women	Attending clinic in University hospital in Japan	Laboratory study	Reduction in PV discharge with SM, Younger patients tended to learn SM, Abnormal vaginal microbiota as frequently seen in SM group.
Medline	Murray et al, 2016	Vaginal pessaries: can an educational brochure help patients to better understand their care?	60 women with stage 2 or 3 symptomatic prolapse	Attending pessary clinic in hospital Australia	Prospective study with pretest-posttest	85% read the SM brochure at 1/52 and 97% at 2/52, Increased satisfaction with SM information given with patient information brochure than with verbally alone, Increased confidence SM with written information, Brochure= increased happiness with information given, increased confidence in SM, increased knowledge re: POP, Increased knowledge re: what a pessary does, increased knowledge re: what to do if a pessary falls out, increased knowledge re: when to use oestrogen, increased knowledge regarding what to do if experiencing discharge, vaginal bleeding, a UTI or urinary leakage.

For peer review only

Medline	Tenfelde et al, 2015	Quality of life in women who use pessaries for longer than 12 months.	56 pessary using women	Attending tertiary clinic in USA	Observational cross-sectional study	Most SM women removed pessary daily (40%) or a few times per week (40%). 20% Removed pessary monthly or greater. 44% of women self-managing pessary.
Medline	Manchana, 2011	Ring pessary for all pelvic organ prolapse.	100 women with symptomatic POP	Attending outpatient gynaecology clinic in Thailand	Retrospective case note review	Only 25 women (40%) could SM by themselves or with caregivers assistance. 4 of 25 SM women (16%) and 23 of 37 non SM women (62%) had adverse events e.g. discharge. Self-care protocol given as a reason for pessary discontinuation. The ring pessary is easy to teach patient to SM.
Embase	Ma et al, 2021	Vaginal pessary treatment in women with symptomatic pelvic organ prolapse: a long-term prospective study.	312 women with symptomatic prolapse receiving pessary management	Attending tertiary referral centre in China	Prospective observational study	Women were advised to perform weekly self-care. Inability to SM a potential factor in discontinuing pessary management.
Embase	*Chen et al, 2020a	The pessary fits, now what? clinical factors associated with pessary self-management Vs. Clinic management.	218 long term pessary users (excluded those using as interim before surgery or did not complete trial)	Tertiary urogynaecology clinic USA	Retrospective notes review	Of 218 women, 159 were self-managing (73%). Increased age, increased BMI, increased prolapse stage, type of pessary and raised POPQ score for Ba, Bp, C and GH increased the odds of office management.

For peer review only

Embase	Holubyeva, 2021	Rates of pessary self-care and the characteristics of patients who perform it.	Notes of 580 women receiving pessary care	Tertiary care centre in USA	Retrospective notes review	31% (174/563) chose to SM. Patients performing SM were seen less frequently (4-6 monthly) whereas non-SM women were seen 1-3 monthly. Less SM experienced PVB (16% vs 26%) and vaginal erosions (10% vs 24%). Patients in SM group significantly younger. Post-menopausal women more likely to want office care. Women with lower stage prolapse (1-2) more likely to SM. SM women more likely to be fitted with a ring pessary or ring with support whereas office-based care women more likely to be fitted with a gell-horn or ring with support. Women who opted not to SM had a slightly higher BMI, increased rates of arthritis and cognitive impairment, however none of this was statistically significant.
Embase	*Chen et al, 2020b	Pessary self-management vs. clinic management: Is one more likely to result in surgery?	218 long term pessary users (excluded those using as interim before surgery or did not complete trial)	Tertiary urogynaecology clinic USA	Retrospective notes review	Of 159 SM women, 30 (19%) had surgery and of 59 office managed women, 20 (35% had surgery.

For peer review only

Embase	Chien et al, 2020	Long-term outcomes of self-management gellhorn pessary for symptomatic pelvic organ prolapse	Notes of 93 women using gell-horn pessary	Tertiary urogynecology unit in Taiwan	Retrospective notes review	Of 421 women referred for gell-horn SM teaching, 147 elected to proceed-note to self, unsure whether that was just SM or gell-horn itself. Patients taught how to deal with and maintain the gell-horn pessary at home. Of 421 women referred for gell-horn SM teaching, 147 elected to proceed-note to self, unsure whether that was just SM or gell-horn itself. At study end point, 48% had used gell-horn continuously with a mean duration of 50 months. 53 (57%) of women reported AEs. 37 (40%) had vaginal pain or discomfort with gell-horn in situ or during removal or insertion. Elected for surgery, inconvenience, discomfort while pessary in situ or being removed or inserted or resolution of pessary symptoms. Told to wash and reinsert when bathing.
Embase	*Lammers et al, 2019	Does monthly self-management of vaginal ring pessaries reduce complication rates?	Notes of 75 women taught to SM their pessary	Tertiary referral urogynaecology unit in Australia	Retrospective notes review	5 women had vaginal erosions (7%) and 3 women had minor complications (4%) which resolved with temporary pessary removal. Overall complication rate was 12% which compares with 56% of non SM women (complications classed as PVB or discharge. 5 women had vaginal erosions (7%) and 3 women had minor complications (4%) which resolved with temporary pessary removal. Overall complication rate was 12% which compares with 56% of non SM women

For peer review only

						(complications classed as PVB or discharge). 4 (7%) proceeded to surgery.
Embase	*Morcuende et al, 2018	Pessary treatment of pelvic organ prolapse: Self care vs. office-based care.	275 women with POP who had pessary management for longer than 3 months	Hospital in America	Retrospective records review	SM women seen yearly or PRN compared with 2-3 monthly. SM group were younger, more sexually active, had fewer comorbidities and fewer stage 3 and 4 prolapse. Women in the office care group were more likely to have a space occupying pessary or have required a change in size or type of pessary before. Office care was associated with increased rate of erosions in each group despite adjusting for age, comorbidities, POP stage and pessary type. Non SM women more likely to have vaginal erosions.
Embase	*Hooper et al, 2018	Cube pessary use and quality of life in women with pelvic organ prolapse	25 women with symptomatic POP fitted with a pessary	Hospital in America	Prospective, observational multi-site study	12 or 25 women were able to self-manage cube pessary. Cube pessary improved QOL and vaginal symptoms
Embase	Chan et al, 2019	What are the clinical factors that are predictive of persistent pessary use at 12 months?	152 New pessary fittings in patients	Tertiary care urogynaecology clinic in Canada	Retrospective records review	Mode of care: self-care (65% n=98) nurse-care (26% n=39). Protocol advised women not to leave the pessary in situ for longer than 3/12. Many women performed self-removal and cleaning on a daily or weekly basis. Nurse vs Self-care not statistically significant to continuation of pessary use at 12/12.
Embase	*Pizarro-Berdichevsky et al, 2016	Pessary use in patients with symptomatic pelvic organ prolapse-12	133 women who have used a pessary for a year	Hospital in Chile	Prospective questionnaire/notes study	68% of patients were able to SM. Inability to SM linked with drop out.

For peer review only

		month prospective study of factors predictive of subjective improvement	or longer POP stage 2+			
Embase	*Ibrahim et al, 2015	Evaluation of pessary service for women with pelvic organ prolapse (POP)	104 women attending pessary clinic	Different hospitals in Scotland	Questionnaire study	9% of women attending the pessary clinic would consider SM. A common reason for not wanting to SM was lack of confidence.
Embase	Khaja and Freeman, 2014	How often should shelf/Gellhorn pessaries be changed? A survey of IUGA urogynaecologists	322 Members of IUGA or BSUG	International	Electronic questionnaire study	Self-care usually acceptable with ring pessary. 86% (n=277) do not believe shelf or gell-horn pessaries are suitable for self care.

For peer review only

Embase	Kearney and Brown, 2014	Self-management of vaginal pessaries for pelvic organ prolapse	134 pessary using women	Tertiary urogynaecology clinic in Cambridge	Quality improvement and evaluation	73% of women who agreed to commence SM were SM at 6/12. No difference in ability to SM depending on size of pessary. SM women had increased level of comfort with pessary changes. SM women reported increased convenience. SM women reported increased access to help. SM women reported increased feeling of support. SM women had increased level of comfort with pessary changes. 1 complication of increased discharge advised to remove the pessary less frequently.<br>Reasons for pessary removal n=45<br>• 6/12 change (36% n=16)<br>• To clean (31% n=14)<br>• To aid defecation (11% n=5)<br>• Discomfort (n=10 22%)<br>• To have sex (n=6 13%)<br>• Smear or other procedure (n=2 4%)<br>• Holiday (n=1 2%)<br><br>47 women declined SM due to:<br>• Physical barriers (33% and n=15)<br>• Prefers Dr led care (17% n=8)<br>• Clinician found pessary removal/insertion difficult (13% n=6)<br>• Nature of intervention (11% n=5)<br>• Too painful (9% n=4)<br>• Emotional barrier (9% n=4)<br>• Feels too old (6% n=3)<br>• Poor cognitive ability (6% n=3)

For peer review only

						• Travel arrangements to return for teach SM 4% n=2)<br>• Personal circumstance (4% n=2)<br><br>24 women (27%) stopped SM by 6/12 due to:<br>• Manual dexterity (n=9 38%)<br>• PVB (n=1 4%) and therefore Dr led care<br>• Switched to shelf pessary (n=1 4%) and therefore Dr led care<br>• Discontinued pessary management (n=11 46%)<br>• Opted for Surgical management (n=2 8%).<br>Frequency of pessary removal and insertion ranged from twice a week to once every 6 months. More women were able/willing to SM with a ring than sieve pessary. 97% of SM women plan to use pessary long term compared with 70% in Dr care group. At 6/12 of SM 17 of 25 women (68%) were no longer considering surgery. Cost saving for Trust and commissioners. The teaching package was delivered by a specialist women's health physiotherapist. Recruiting a physiotherapist to this project was not something that we had originally considered as we had initially seen this as a specialist nursing role. However, we believe that by having a physiotherapist (a role which

For peer review only

						traditionally focuses on rehabilitation and not medical intervention) at the heart of the service, we have been able to develop something which is truly focussed on empowering the patient to manage their own condition away from a healthcare setting. During a single 45-minute appointment, informed consent was obtained and the woman was shown and supervised in pessary change. This face to face training was supplemented with written information and an online teaching video. Feedback from the focus group on the information leaflets was used to inform the structure of the teaching video, with increased emphasis placed on simple diagrammatic presentation of prolapse and pessary in situ.

For peer review only

Embase	Nemeth et al, 2013.	The cube pessary: An underestimated treatment option for pelvic organ prolapse? Subjective 1-year outcomes.	87 women with stage 2 or 3 prolapse	Urogynaecology clinic in teaching hospital Hungary	Prospective case review	85.5% of women rated SM with a cube pessary as very easy or easy
Embase	*Jacobs and Banks, 2010	Pilot study: Pessary use in Bronx women. Would increasing the practice of self-changing pessaries have potential to positively impact body image?	25 women attending a pessary follow-up clinic	Urogynaecology clinic in hospital USA	Questionnaire study	Despite a high level of function in everyday life, only 50% of women felt capable to SM. 1 of 25 women SM. Only a further 2 felt able to attempt. Only 4 would be willing to try. 12 replied that they would like to change their pessary but felt they couldn't. Body image and function scores equivalent to non pessary users therefore these factors do not appear to link with willingness to SM.
CINAHL	Goh et al, 2020	An audit of vaginal support pessaries for pelvic organ prolapse in western Uganda	93 women with stage 3 or 4 prolapse	Fistula/prolapse camp in Western Uganda	Audit	All women (93) taught to SM. 65 trialled pessary for 2 days successfully and were able to SM. Taught to remove once weekly, wash, dry and reinsert. 54 (83%) described SM as easy and had increased confidence in being able to do this after one tutorial.
CINAHL	*Daneel et al, 2016	Does monthly self-removal of vaginal ring pessaries for stress urinary incontinence/ prolapse reduce	74 women trained in self-management of ring for POP/SUI	Tertiary urogynaecology clinic in Australia	Audit	At FU, 66% of women still SM. Complication rate for long term SM women was 10%. Lower complication rate for SM women compared with non-SM women in other studies. Patient wanted surgery, unable to SM due to barriers such as obesity, arthritic fingers)

For peer review only

		complication rates? A 5 year audit.				and pessary related complications. Taught to SM by CNS. Women SM rings, Shaatz and gell-horns. Annual FU if SM
CINAHL	Manonai et al, 2018	Vaginal ring pessary use for pelvic organ prolapse: continuation rates and predictors of continued use.	289 women with symptomatic stage 2, 3 and 4 POP	Tertiary centre Thailand	Retrospective chart review	Self-management protocol included general education re: pessary, self-care coaching re insertion, removal and cleaning, written information and a hotline to call with any emergencies. Self-care of a pessary was the only significant factor identified that influenced the continuation rate with pessary management at 3 years. Women who did not require assistance for pessary changes from a healthcare professional or family member were significantly more likely to continue with pessary use.
CINAHL	Storey et al, 2009	Women's experiences with vaginal pessary use.	11 post-menopausal women	Urogynaecology clinic in Canada	Narrative inquiry was used to conduct face-to-face semi-structured interviews.	Of 11, only 3 opted to SM. Women referred to being older as a reason not to want to SM. The women who SM reported that they chose it due to confidence in their ability to do so and desire for flexibility in when and how they used their pessary. Reasons for not wanting to SM were discomfort 'touching themselves', lack of confidence with removal and insertion. Despite this, the women who declined SM showed high levels of confidence in dealing with daily challenges such as adjusting their pessary after opening their bowels. Women described clinic

For peer review only

						visits as psychological and emotional support as well as physical and pessary care and viewed them as social outings. Possible reason not to want to SM.
Handsearch	Meriwether et al, 2015a	The effect of hydroxyquinoline-based gel on pessary associated BV	184 women attending pessary clinic	2 tertiary care centres in USA	Multicentre RCT	Frequency of pessary use or removal did not affect the groups in terms of BV or vaginal symptoms at 2 weeks or 3 months. 34 out of 136 women (25%) who reported frequency of SM, removed at least once a day.
Handsearch	Meriwether et al, 2015b	Sexual function and pessary management among women who are using a pessary for pelvic floor dysfuction	127 women presenting for a pessary fitting	Two tertiary care centres in USA	Secondary analysis of a randomized trial of new pessary users	Most common reason for r/o pessary, partner could feel during sex (20%) No women reported vaginal discharge or odour as reason for r/o pessary prior to sex. Daily pessary removal association with being sexually active at 3 months but did not improve sexual function or body image scores. For non-sexually active women, daily pessary removal associated with increased scores on partner related domain.
Handsearch	Tam et al, 2019	The effect of time interval of vaginal ring pessasry replacement for pelvic organ prolapse on complication and patient satisfaction a randomised controlled trial	60 women with stage 1-4 POP who opted for pessary management	Tertiary urogynecology center in Hong Kong	Double-blinded, randomized controlled trial	Self care of ring pessary performed by 2 out of 60 women. Many women refused to learn self-care.

For peer review only

Handsearch	Fregosi et al, 2018	Changes in the Vaginal Microenvironment as Related to Frequency of Pessary Removal	137 new pessary users attending pessary clinic	2 tertiary care centres in USA	Secondary analysis of RCT	34 (25%) removed the pessary daily, 54 (39%) at least weekly, and 49 (36%) less often than once weekly. Women who removed the pessary less often than weekly were older, using more hormone therapy, and more likely to have BV at baseline. Women who removed their pessaries less often than once weekly had an increased prevalence of anaerobes at 3 months, but no difference in vaginal symptoms or pessary satisfaction.
Handsearch	Clemons et al, 2004a	Patient characteristics associated with continued pessary use versus surgery after one year	67 women satisfied with pessary at 2 months for stage 2 or more POP	Urogynaecology clinic in USA	Prospective observational study	Ability to change pessary not associated with continued pessary use or decision to opt for surgical management. SM women FU 6-12 monthly, otherwise 2-3 monthly.
Handsearch	Clemons et al, 2004b	Patient satisfaction and changes in prolapse and urinary symptoms in women who were fitted successfully with a pessary for pelvic organ prolapse	73 women with stage 2 or more prolapse successfully fitted with a pessary	Urogynaecology clinic in USA	Prospective observational study	21 women (29%) were taught to insert and remove their pessary. All of the women taught to SM used ring pessaries because gell-horn are too difficult to SM.
Handsearch	Kuhn et al, 2008	Sexual organ function in patients with symptomatic prolapse: are pessaries helpful?	73 women with stage 2 or more prolapse	Tertiary referral centre in Switzerland	Prospective observational study	(Cube pessary) Patients received advice to remove the pessary as required for sexual intercourse

For peer review only

Handsearch	Bugge et al, 2013	Vaginal pessaries for pelvic organ prolapse and urinary incontinence: a multiprofessional survey of practice	678 members of RCOG, the Association for Continence Advice and the Association of Chartered Physiotherapists in Women's Health in the UK	Members of RCOG the Association for Continence Advice and the Association of Chartered Physiotherapists in Women's Health in the UK.	Electronic questionnaire	17.7% (n=84) UK HCP recommend self-care following fitting. 4.1% (n=21) report patient responsible for pessary aftercare at organisation
Handsearch	Thys et al, 2020	Effect of pessary cleaning and optimal time interval for follow-up a prospective cohort study	163 women with stage 2 or more primary prolapse fitted with pessary	Outpatient clinic at 3 teaching hospitals. The Netherlands.	Prospective cohort study	SM performed by 45.2% of women after one year. Patients who performed SM were younger, more frequently diagnosed with atrophy, reported less concomitant SUI and were more often sexually active. No difference between SM and non SM women in vaginal pain, discharge or irritation. No evidence for need to check asymptomatic patients performing SM with a silicone ring. SM more often sexually active including vaginal penetration.
Handsearch	Hanson et al, 2006	Vaginal pessaries in managing women with pelvic organ prolapse and urinary incontinence: patient characteristics and factors contributing to success	1216 women referred to nurse led pessary clinic	Nurse led pessary clinic in Canada	Retrospective notes review	All patients were encouraged to learn to remove and reinsert the pessary weekly in order to clean the pessary and maintain their independence. Many patients were able to remove and clean their own pessary. Gell-horn and cube pessaries are the most difficult to remove, however a number of women were able to remove their gell-horn.

For peer review only

Handsearch	Abdool et al, 2011	Prospective evaluation of outcome of vaginal pessaries versus surgery in women with symptomatic pelvic organ prolapse	554 women with symptomatic prolapse treated with either surgery or pessary	Specialist urogynaecology unit UK	Prospective questionnaire study	Many women preferred not to handle the pessary therefore a ring pessary was inserted for all sexually active women to avoid this.
Handsearch	Bai et al, 2005	Survey of the characteristics and satisfaction degree of the patients using a pessary	104 women fitted with a pessary and available for FU	Urogynaecology clinic South Korea	Retrospective review of notes and patient completed questionnaire	The ring pessary is easy to wear and remove and effective. While the donut pessary is hard for a patient to remove by herself because the pessary does not have a notch. 19.1% of women removed their pessary (80% due to it slipping or discomfort)
Handsearch	Clemons et al, 2004c	Risk factors associated with an unsuccessful pessary fitting trial in women with pelvic organ prolapse	100 consecutive women with symptomatic POP fitted with a pessary	Urogynaecology clinic in USA	Prospective observational study	28 women (38%) were taught how to remove, clean and reinsert their pessary (ring or gell-horn pessary).
Handsearch	Sulak et al, 1993	Vaginal pessaries and their use in pelvic relaxation	101 women fitted with a pessary who returned for follow-up	Gynaecology clinic in USA	Retrospective review	5 of the 50 pessary users (10%) mentioned occasional difficulty with removal of the pessary (gell-horn). Another 5 had to have the pessary removed periodically by a nurse. For gell-horn, all patients (n=96) were advised to remove once or twice a week and leave it out overnight n=35 (70% removed the pessary at least once a week. The remainder removed it at least once every 6 weeks apart from one woman who had it in place for 6/12 without issue

Handsearch	*Ramsay et al, 2011	Long-term outcomes of pessary use in women with pelvic organ prolapse	429 women who underwent a pessary trial	Urogynaecology clinic in Hospital in Canada	Retrospective case notes review	Pessary SM associated with prolonged pessary use. 66% of women were able to SM.
Handsearch	Brown et al, 2016	Defining patient's knowledge and perceptions of vaginal pessaries for prolapse and incontinence	254 women urogynaecology attendees	Urogynaecology clinic at an academic medical centre in the USA	Convenience sample questionnaire study	Few women indicted concerns about pessary management for example that they would not be able to remove the pessary independently (n=20 16%).
Handsearch	Wu et al, 1997	A simplified protocol for pessary management	110 women receiving pessary management	Clinic in Canada	Prospective modified pessary care protocol followed and long term data collected at follow-up appointments	Ring pessary first choice as similar size and shape to diaphragm which patients find easy to insert and remove Most patients chose not to SM their pessary and return for regular office visits for pessary changes
Handsearch	Sarma et al, 2009	Long-term vaginal ring pessary use: discontinuation rates and adverse events	273 women fitted with a ring pessary in a tertiary urogynaecology clinic	Clinic in Australia	Retrospective notes review	All women who had reasonable manual dexterity were offered the option of self-management. Self-managing women were then seen 12 monthly.
Handsearch	Cundiff et al, 2000	A survey of pessary users by members of the American Urogynaecology Society	359 members of AUGS	AUGS members	Questionnaire study	53% (190) doctors reported teaching all patients to change their own pessary. 45% (162) reserve self-management for a subset of women using support pessaries

Monday, Octob

	Limiters/Expanders	Last Run Via
nagement or self care)	Expanders - Apply equivalent subjects<br>Search modes - Boolean/Phrase	Interface - EBSCOhost Research Databases<br>Search Screen - Advanced Search<br>Database - CINAHL Plus

Embase <1974 to 2021 May 07>

1	self care/	65562
2	vagina pessary/	3004
3	1 and 2	44

For peer review only

Ovid MEDLINE(R) <1946 to May Week 1 2021>

1	Pessaries/	1578
2	Self Care/ or Self-Management/	37957
3	1 and 2	16

For peer review only

APA PsycInfo <1806 to May Week 4 2021>

1	exp Self-Management/	7368
2	exp Self-Care/	2977
3	1 or 2	10087
4	pessary.mp.	9
5	pessaries.mp.	14
6	4 or 5	17
7	3 and 6	0

For peer review only

Preferred Reporting Items for Systematic reviews and Meta-Analyses extension for Scoping Reviews (PRISMA-ScR) Checklist

SECTION	ITEM	PRISMA-ScR CHECKLIST ITEM	REPORTED ON PAGE #
**TITLE**			
Title	1	Identify the report as a scoping review.	1
**ABSTRACT**			
Structured summary	2	Provide a structured summary that includes (as applicable): background, objectives, eligibility criteria, sources of evidence, charting methods, results, and conclusions that relate to the review questions and objectives.	2-3
**INTRODUCTION**			
Rationale	3	Describe the rationale for the review in the context of what is already known. Explain why the review questions/objectives lend themselves to a scoping review approach.	5
Objectives	4	Provide an explicit statement of the questions and objectives being addressed with reference to their key elements (e.g., population or participants, concepts, and context) or other relevant key elements used to conceptualize the review questions and/or objectives.	5
**METHODS**			
Protocol and registration	5	Indicate whether a review protocol exists; state if and where it can be accessed (e.g., a Web address); and if available, provide registration information, including the registration number.	3 and 5
Eligibility criteria	6	Specify characteristics of the sources of evidence used as eligibility criteria (e.g., years considered, language, and publication status), and provide a rationale.	Image 1
Information sources*	7	Describe all information sources in the search (e.g., databases with dates of coverage and contact with authors to identify additional sources), as well as the date the most recent search was executed.	Page 5 and published protocol
Search	8	Present the full electronic search strategy for at least 1 database, including any limits used, such that it could be repeated.	Supplementary material
Selection of sources of evidence†	9	State the process for selecting sources of evidence (i.e., screening and eligibility) included in the scoping review.	Page 5 and published protocol
Data charting process‡	10	Describe the methods of charting data from the included sources of evidence (e.g., calibrated forms or forms that have been tested by the team before their use, and whether data charting was done independently or in duplicate) and any processes for obtaining and confirming data from investigators.	Page 5 and published protocol
Data items	11	List and define all variables for which data were sought and any assumptions and simplifications made.	Published protocol
Critical appraisal of individual	12	If done, provide a rationale for conducting a critical appraisal of included sources of evidence; describe	Page 7 and published protocol

St. Michael's
Inspired Care.
Inspiring Science.

For peer review only - http://bmjopen.bmj.com/site/about/guidelines.xhtml

SECTION	ITEM	PRISMA-ScR CHECKLIST ITEM	REPORTED ON PAGE #
sources of evidence§		the methods used and how this information was used in any data synthesis (if appropriate).	
Synthesis of results	13	Describe the methods of handling and summarizing the data that were charted.	Page 5 and published protocol
**RESULTS**			
Selection of sources of evidence	14	Give numbers of sources of evidence screened, assessed for eligibility, and included in the review, with reasons for exclusions at each stage, ideally using a flow diagram.	Page 6 and image 2
Characteristics of sources of evidence	15	For each source of evidence, present characteristics for which data were charted and provide the citations.	Supplementary material
Critical appraisal within sources of evidence	16	If done, present data on critical appraisal of included sources of evidence (see item 12).	Image 3
Results of individual sources of evidence	17	For each included source of evidence, present the relevant data that were charted that relate to the review questions and objectives.	Supplementary material
Synthesis of results	18	Summarize and/or present the charting results as they relate to the review questions and objectives.	6-14
**DISCUSSION**			
Summary of evidence	19	Summarize the main results (including an overview of concepts, themes, and types of evidence available), link to the review questions and objectives, and consider the relevance to key groups.	14
Limitations	20	Discuss the limitations of the scoping review process.	14-15
Conclusions	21	Provide a general interpretation of the results with respect to the review questions and objectives, as well as potential implications and/or next steps.	15
**FUNDING**			
Funding	22	Describe sources of funding for the included sources of evidence, as well as sources of funding for the scoping review. Describe the role of the funders of the scoping review.	16

JBI = Joanna Briggs Institute; PRISMA-ScR = Preferred Reporting Items for Systematic reviews and Meta-Analyses extension for Scoping Reviews.

* Where *sources of evidence* (see second footnote) are compiled from, such as bibliographic databases, social media platforms, and Web sites.

† A more inclusive/heterogeneous term used to account for the different types of evidence or data sources (e.g., quantitative and/or qualitative research, expert opinion, and policy documents) that may be eligible in a scoping review as opposed to only studies. This is not to be confused with *information sources* (see first footnote).

‡ The frameworks by Arksey and O'Malley (6) and Levac and colleagues (7) and the JBI guidance (4, 5) refer to the process of data extraction in a scoping review as data charting.

§ The process of systematically examining research evidence to assess its validity, results, and relevance before using it to inform a decision. This term is used for items 12 and 19 instead of "risk of bias" (which is more applicable to systematic reviews of interventions) to include and acknowledge the various sources of evidence that may be used in a scoping review (e.g., quantitative and/or qualitative research, expert opinion, and policy document).

From: Tricco AC, Lillie E, Zarin W, O'Brien KK, Colquhoun H, Levac D, et al. PRISMA Extension for Scoping Reviews (PRISMAScR): Checklist and Explanation. Ann Intern Med. 2018;169:467–473.

St. Michael's
Inspired Care.
Inspiring Science.

BMJ Open

What is known from the existing literature about self-management of pessaries for pelvic organ prolapse? A scoping review.

Journal:	*BMJ Open*
Manuscript ID	bmjopen-2021-060223.R1
Article Type:	Original research
Date Submitted by the Author:	30-Apr-2022
Complete List of Authors:	Dwyer, Lucy; Manchester University NHS Foundation Trust, The Warrell Unit; The University of Manchester School of Health Sciences, Division of Nursing, Midwifery and Social Work<br>Dowding, Dawn; University of Manchester<br>Kearney, R; Manchester Academic Health Science Centre; University of Manchester
**Primary Subject Heading**:	Obstetrics and gynaecology
Secondary Subject Heading:	Health services research, Nursing
Keywords:	Urogynaecology < UROLOGY, GYNAECOLOGY, STATISTICS & RESEARCH METHODS

SCHOLARONE™
Manuscripts

I, the Submitting Author has the right to grant and does grant on behalf of all authors of the Work (as defined in the below author licence), an exclusive licence and/or a non-exclusive licence for contributions from authors who are: i) UK Crown employees; ii) where BMJ has agreed a CC-BY licence shall apply, and/or iii) in accordance with the terms applicable for US Federal Government officers or employees acting as part of their official duties; on a worldwide, perpetual, irrevocable, royalty-free basis to BMJ Publishing Group Ltd ("BMJ") its licensees and where the relevant Journal is co-owned by BMJ to the co-owners of the Journal, to publish the Work in this journal and any other BMJ products and to exploit all rights, as set out in our licence.

The Submitting Author accepts and understands that any supply made under these terms is made by BMJ to the Submitting Author unless you are acting as an employee on behalf of your employer or a postgraduate student of an affiliated institution which is paying any applicable article publishing charge ("APC") for Open Access articles. Where the Submitting Author wishes to make the Work available on an Open Access basis (and intends to pay the relevant APC), the terms of reuse of such Open Access shall be governed by a Creative Commons licence – details of these licences and which Creative Commons licence will apply to this Work are set out in our licence referred to above.

Other than as permitted in any relevant BMJ Author's Self Archiving Policies, I confirm this Work has not been accepted for publication elsewhere, is not being considered for publication elsewhere and does not duplicate material already published. I confirm all authors consent to publication of this Work and authorise the granting of this licence.

For peer review only

What is known from the existing literature about self-management of pessaries for pelvic organ prolapse? A scoping review.

Dwyer. L[1,2], Dowding. D [2] and Kearney. R[1,3]

Affiliations:

[1] Saint Mary's Hospital, Manchester University NHS Foundation Trust, Manchester Academic Health Science Centre, Hathersage Road, Manchester M13 9WL, UK

[2]Division of Nursing, Midwifery and Social Work, School of Health Sciences, Faculty of Biology, Medicine and Health, University of Manchester, Oxford Road, Manchester, M13 9PL

[3] Institute of Human Development, Faculty of Medical & Human Sciences, University of Manchester, Oxford Road, Manchester, M13 9PL

Corresponding author: Lucy Dwyer

lucy.dwyer@postgrad.manchester.ac.uk 0161 276 6910

The Warrell Unit, Saint Mary's Hospital,

Manchester University NHS Foundation Trust

Hathersage Road

Manchester

M13 9WL

Dawn Dowding dawn.dowding@manchester.ac.uk

Rohna Kearney rohna.kearney@mft.nhs.uk

Abstract word count: 281

Main article word Count: 4046

Key words: Pessary, self-management, self-care, prolapse, scoping,

For peer review only - http://bmjopen.bmj.com/site/about/guidelines.xhtml

What is known from the existing literature about self-management of pessaries for pelvic organ prolapse? A scoping review.

ABSTRACT

Objectives

Pelvic organ prolapse (POP) can be managed with a pessary. However, regular follow-up may deter women due to the inconvenience of frequent appointments, as well as preventing autonomous decision making. Pessary self-management, may be a solution to these issues. However, there remains a number of uncertainties regarding pessary self-management. This scoping review aims to map available evidence about pessary self-management to identify knowledge gaps providing the basis for future research.

Design

Scoping review as detailed in the review protocol.

Data sources

A search of MEDLINE, CINAHL, EMBASE and PsycInfo databases and a hand search were undertaken during May 2021 to identify relevant articles using the search terms 'pessary' and 'self-management' or 'self-care'.

Data extraction and synthesis

Data relevant to pessary self-management was extracted and the Mixed Methods Appraisal Tool used to assess empirical rigour. Thematic analysis was performed to evaluate the results.

Results

The database search identified 82 publications. After duplicates and articles not meeting the inclusion and exclusion criteria were removed, there were 23 eligible articles. A hand search revealed a further 19 articles, resulting in a total of 42 publications.

Findings relevant to pessary self-management were extracted and analysed for the emergence of themes. Recurrent themes in the literature were; the characteristics of self-managing women; pessary care; factors associated with decision making about self-management; teaching self-management and cost benefit.

For peer review only - http://bmjopen.bmj.com/site/about/guidelines.xhtml

Conclusions

Pessary self-management may offer benefits to some women without increased risk. Some women do not feel willing or able to self-manage their pessary. However, increased support may help women overcome this. Further in-depth exploration of factors which affect women's willingness to self-manage their pessary is indicated to ensure better understanding and support as available for other conditions.

ARTICLE SUMMARY

Strengths and limitations of this study

- The scoping review was undertaken following systematic and rigorous established guidelines to ensure transparency and reproducibility.
- Critical appraisal of the identified publications enabled identification of methodological limitations within this subject area
- Studies not published in English were excluded which increases the risk of cultural bias in the findings

For peer review only

What is known from the existing literature about self-management of pessaries for pelvic organ prolapse? A scoping review.

INTRODUCTION

Mechanical pessaries can effectively reduce symptoms of pelvic organ prolapse and therefore offer an effective alternative to surgery[1] However, the regular pessary follow-up required deters some women from this management option[2-4]. It has been suggested pessary self-management may reduce the frequency of pessary follow-up required. Moreover, it offers women the opportunity to make autonomous decisions about when and how to use their pessary. Nevertheless, there remains a number of uncertainties about pessary self-management including the potential benefits and risks and requirements of self-management teaching and follow-up care[5]. This scoping review aims to map available evidence about pessary self-management to identify knowledge gaps[6] providing the basis for future research.

METHODS

As detailed in the published protocol (Dwyer et al, accepted by BMJ Open-Dec 21), the scoping review was conducted utilising the Joanna Briggs Institute scoping review methodology[7] and reported in accordance with Preferred Reporting Items for Systematic reviews and Meta-Analyses extension for Scoping Reviews PRISMA-ScR guidelines[8] (Supplementary material 1). The review was registered with The Open Science Framework (OSF) (DOI 10.17605/OSF.IO/DNGCP). Between 05/05/21 and 07/05/21 a search of MEDLINE, CINAHL, EMBASE and PsycInfo was undertaken to identify relevant articles which met the eligibility criteria (Image 1) using the search terms 'pessary' and 'self-management' or 'self-care' (see supplementary material 2-5). Hand searches for relevant and eligible publications not identified during the search were undertaken throughout May 2021.

A hand search of the reference list of non-original research identified during the search but excluded, was conducted for additional publications which met the inclusion and exclusion criteria. Data relevant to the topic of pessary self-management was extracted and critical appraisal of all included publications undertaken using the Mixed Methods Appraisal Tool (MMAT) [9].

PATIENT AND PUBLIC INVOLVEMENT

For peer review only - http://bmjopen.bmj.com/site/about/guidelines.xhtml

Members of the public and pessary users have not directly been involved with development of the review protocol or process. However, the need for research exploring pessary self-management was highlighted by The James Lind Alliance (JLA) Priority Setting Partnership for pessary and prolapse[10]. Several women with experience of pessaries participated in this partnership either as members of the steering group, by attending the consensus workshop or completing questionnaires. Understanding more about self-management was ranked third out of 20 priorities by the JLA Priority Setting Partnership[10]. The topic of the scoping review has therefore previously been identified and prioritised by patients and members of the public.

RESULTS

The database search identified 82 publications. After duplicates and articles not accessible in the English language were removed, there were 64 remaining publications. After reviewing the identified articles in accordance with the inclusion and exclusion criteria (Image 1), there were 23 eligible articles. A hand search of reference lists from excluded non-original research papers, revealed a further 19 articles, resulting in a total of 42 publications (Image 2, Supplementary material 6). Ten of the 42 publications included were solely available as an abstract which prevented thorough understanding and appraisal of both the findings and methodology.

For peer review only

For peer review only - http://bmjopen.bmj.com/site/about/guidelines.xhtml

Table 1-Summary of included studies

**Total number of publications included**	42	
**Continent where research based**		
Africa	1	
Asia	7	
Australasia	4	
Europe	7	
North America	21	
South America	1	
International	1	
**Publication date- Median (Range)**	2015	1993-2021
**Publication type**		
Abstract	10	
Journal article	32	
**Study design**		
Audit	2	
Case note review	14	
Cross sectional	1	
Laboratory study	1	
Observational	17	
Pre-test Post test	1	
Qualitative	1	
Quality improvement evaluation	1	
Randomised controlled trial	2	
Secondary analysis of RCT	2	

For peer review only - http://bmjopen.bmj.com/site/about/guidelines.xhtml

As demonstrated in Table 1, the 42 studies included in the review were conducted across all six populated continents. Four (10%) took place in the UK. Half of the eligible studies took place in the USA. Only six (14%) of the eligible studies included in the review were interventional and two of these were secondary analysis of another included publication. A significant majority of the included studies were observational, most being conducted via case note review or questionnaires. A number of included publications report findings from the same sample of women as highlighted in the supplementary material (6). These findings have been reported separately due to the different aims of each publication.

Critical appraisal of the evidence using the MMAT (Image 3) reveals a number of recurring methodological limitations across the evidence base. Firstly, due to the extent of retrospective case note reviews included, the quality of measurements is unlikely to be good due to the lack of a standardised protocol to record details of the pessary fitting and subsequent follow-up[11]. A further limitation is data completeness, due to both high levels of attrition within some of the randomised controlled trials included and a low response rate in many of the quantitative descriptive studies. This may introduce bias to the findings if those who are the most or least satisfied with pessary management are more or less inclined to respond or continue with study participation[11]. Only a small number of the quantitative non-randomised studies included, accounted for confounding variables within their findings by performing stratification, multivariate analysis or matching[11]. In the majority of studies that did not account for confounding variables the validity of the findings is questionable as various factors such as age, hormonal replacement status and prolapse stage could all have influenced the results. A further recurring limitation identified while appraising quantitative descriptive study was the lack of representativeness amongst study participants, whether pessary using women or healthcare professionals. Many studies used convenience samples whether only including pessary users who attended follow-up or healthcare professionals who were members of specialist organisations. Therefore, this may limit the generalisability of study findings[11]. Three of the included publications were surveys conducted amongst pessary practitioners [12-14]. It is acknowledged that data collected through self-reporting has a high risk of bias whether due to recall or social desirability bias [15]. However, these publications offer valuable insight into how healthcare professionals perceive they are or should be, delivering pessary care, therefore they have been included for this reason.

Findings relevant to pessary self-management were extracted from the included publications and analysed for the emergence of themes. Recurrent themes in the literature include the characteristics of self-managing women; pessary care; factors associated with decision making about self-management; teaching self-management and cost benefit.

For peer review only - http://bmjopen.bmj.com/site/about/guidelines.xhtml

Characteristics of self-managing women

The extent of pessary self-management is difficult to gauge, though it appears to vary greatly throughout the world. Estimates vary from 18% of UK pessary practitioners to 53% of pessary practitioners in the USA offering pessary self-management[13, 14]. Reasons for this variation are unclear but could be due to differences in care provision possibly due to associated costs of care. It is therefore possible those living in a country with high healthcare costs, may be more willing to self-manage to reduce the costs of attending follow-up appointments.

Several characteristics were associated with women being more likely to self-manage, including being younger[16-23], being pre-menopausal[23], being sexually active[18, 19, 21], having a higher level of education[18], prolonged pessary use[24], having fewer co-morbidities[17-19, 22, 23], a lower stage of prolapse (one or two)[19, 23], a smaller genital hiatus[22] and being diagnosed with atrophy[21].

Age differences between women who self-manage or receive clinician led care may be due to generational attitudes to pessary use or touching one's genitals, or the value associated with perceiving a clinic appointment as a social event with associated psychological and emotional support as proposed by Storey, Aston [16]. Conversely, older women or those with comorbidities may be less likely to self-manage due to a perception of, or actual increased difficulty. However, this has not yet been established.

Space occupying pessaries fill the vaginal cavity and therefore must be removed prior to sexual penetration[25]. Many other pessaries do not need to be removed for penetrative sex[26] However, 70% of women removed their pessary usually or always before sexual activity because their partner could feel the pessary, or due to discomfort[27]. Over half of the women removed the pessary before sexual intercourse after being asked by their partner rather than due to concerns about vaginal discharge or odour[27]. Despite daily pessary removal being associated with sexual activity, this was not correlated with sexual function[27]. Moreover, for women who were not sexually active, frequent pessary removal was associated with an improved relationship with one's partner.

There is disagreement in the literature regarding whether self-management affects the rate of women who choose to continue with pessary management of their prolapse. Self-management has been associated with pessary management continuation[24, 28-30]. Conversely, other studies reported pessary self-management did not affect pessary continuation rates when compared with either nurse or doctor clinician-led care at one year[19, 31, 32]. It is important to note the studies

For peer review only - http://bmjopen.bmj.com/site/about/guidelines.xhtml

where no relationship was established were based upon shorter-term data. Therefore, it is possible this does not allow sufficient time to measure a difference.

Pessary care

There is a wide range of pessaries available to manage pelvic organ prolapse and these are typically classed as either a support or space occupying pessary[25]. Frequently used support pessaries in the UK include ring pessaries, rings with support and shaatz pessaries[13]. There is agreement amongst the literature that support pessaries are suitable for women to self-manage, with ring pessaries being 'acceptable'[12] and 'easy'[33-35] to remove and insert. Wu, Farrell [33] suggest women may feel comfortable performing self-management with a ring pessary as it is a similar size and shape to a diaphragm. Whether there is any relationship between willingness or ability to self-manage a pessary, and previous use of a vaginal device has not previously been explored and research into this may provide important insight. Kearney and Brown [2] report more women were willing or able to self-manage with a ring than sieve pessary. Further research to assess whether this is replicated amongst a larger population of women is indicated.

Commonly used space occupying pessaries include shelf, gell-horn and cube pessaries[13, 25]. These work by providing both structural support and filling the vaginal cavity to prevent vaginal descent[25]. Women using space occupying pessaries are less likely to perform self-management than those using support pessaries[19, 23]. Moreover, Clemons, Aguilar [36] states gell-horn pessaries are too difficult to self-manage, a perception shared by many pessary practitioners[12, 14]. Despite this widespread belief, a number of studies suggest self-management of space occupying pessaries such as shelf or gell-horn pessaries is possible[37, 38], if in some cases more difficult[39]. After self-management teaching, 62% and 47% of women continued to self-manage their gell-horn pessary after one and five years, respectively[38]. This compares similarly to the percentages of women self-managing with other types of pessaries. Bai, Yoon [34] state the donut pessary is difficult for a woman to remove independently, however no rigorous evidence to support this statement is provided. More perplexing is Hanson et al's[39] assertion that cube pessaries are amongst the most difficult pessary to remove. Again, there is no rigorous evidence regarding this statement. Furthermore, according to the manufacturers, it is a requirement to self-manage a cube pessary as it should be removed on a nightly basis [40]. This is due to the increased risk of complications due to the greater surface area covered by the cube pessary and the mild suction[41].

For peer review only - http://bmjopen.bmj.com/site/about/guidelines.xhtml

At present there is a lack of evidence about the required frequency of pessary removal by self-managing women to inform practice. The frequency of pessary removal and insertion will vary in accordance with the reasons for self-management. The most frequently reported frequency of pessary removal and insertion was daily to a couple of times a week[17-19, 32]. A number of protocols provided specific advice regarding frequency of pessary removal. Whether removal each time they bathed[38] or removal and to leave the pessary out overnight once or twice a week[30, 42]. Seventy percent (n=35) of women followed this advice given and removed the pessary at least weekly[42]. One self-managing woman left the gell-horn pessary in situ for six months without removal and experienced no issues. This is unsurprising, as for most UK centres, standard clinician-led care is to follow-up pessaries on a six-monthly basis[43]. On this basis, Kearney and Brown [2] recommended self-managing women remove their pessary at a minimum of six monthly. Chan, Hyakutake [32] advised women not to leave the pessary in situ for longer than 3 months, which mirrored clinician-led care at that organisation. A self-management protocol advising women to remove their pessary on a weekly basis was cited as the reason for pessary discontinuation as women found it too burdensome[35]. While there is no evidence to inform guidance about recommended frequency of pessary removal and insertion, instructing women to remove their pessary very frequently may deter women from self-management but also reduce the autonomy self-management offers.

The majority of studies suggest pessary self-management offers benefits in terms of women's comfort, convenience, perceived access to help and support and feeling of independence[2, 39]. Self-management reduces complications when compared with clinician-led care, with an overall complication rate for self-managing women of 10-16%[35, 37, 44], compared to complication rates of 62% for women receiving clinician-led care[35]. Studies report a lower rate of vaginal erosions for self-managing women[19, 23]. However, no difference was reported between women self-managing or receiving clinician led care regarding vaginal pain, discharge or irritation[21]. Vaginal pain or discomfort was experienced by 40% (n=37) of women during removal or insertion of their gell-horn pessary[38]. As there was no comparison with women receiving clinician led care it is not known whether the pain and discomfort reported by women is comparable to that experienced by women during clinician-led pessary changes.

There is mixed evidence for the effect on vaginal discharge, with one study reporting an increase for one woman who commenced self-management[2] and another an increase in discharge for women who left their pessary in situ continuously[20]. Despite identifiable differences in the vaginal microenvironment associated with frequency of removal, this did not appear to be clinically significant with regards to women's symptoms after three months[45]. It is possible if conducted

For peer review only - http://bmjopen.bmj.com/site/about/guidelines.xhtml

over a longer period, such as six months which is more akin to the typical length of time a pessary is left in situ[43], greater differences might have been identified. Furthermore, due to the frequency of removal the women were grouped into, the findings cannot be generalised to the large population of women who do not remove their pessary at all.

Factors associated with decision making about self-management

In publications which explored willingness to self-manage a pessary, the percentage of willing women varied significantly, from 3-83%[16, 22, 23, 32, 36, 46-49]. Reports of women's levels of ability to self-manage their pessary also differs extensively between different studies, ranging between 40-86%[2, 21, 24, 28, 35, 37, 41, 44, 50]. Furthermore, ability to self-manage a pessary does not appear to be a straightforward concept. Twenty percent of women reported occasional difficulty removing their pessary or required assistance from a healthcare professional[42]. After teaching, 17% of women initially found self-management difficult, however, this reduced significantly with repeated attempts[51]. It is unclear why self-management ability may fluctuate. Manual dexterity was cited as an important aspect of self-management ability[52]. Kearney and Brown [2] found issues with this to be the most common barrier, causing seven percent of women to discontinue self-management after six months.

Three studies explored the reasons why women self-managed their pessary. Mostly women removed or readjusted their pessary when it was slipping or uncomfortable[34], or because they desired flexibility in how and when they used their pessary[16]. After six months of self-management the most frequently cited reason for pessary removal was removal and reinsertion at six months as advised by their clinician (36%, n=16). This was followed by removal for cleaning (31%, n=14), removal to aid defecation (11%, n=5), removal due to discomfort (22%, n=10), removal to have sex (13%, n=6) removal for a smear or other procedure (4%, n=2) and removal before a holiday (2%, n=1). It is possible the reasons for removal may be different if collected over a longer period than six months. However, it offers clinicians some understanding into self-management behaviours of women.

Women who experienced pessary related complications were also less likely to opt for self-management, or to discontinue self-management whether due to patient request[37] experiencing unexpected bleeding, or changing to a pessary not suitable for self-management[2]. Physical barriers were the most cited reason women gave to Kearney and Brown [2] for non-participation in the study. However, further detail was not provided therefore it is unclear exactly what these physical

For peer review only - http://bmjopen.bmj.com/site/about/guidelines.xhtml

barriers were. Another physical barrier to self-management reported by 6% of women was feeling ‘too old’. Whether age as a barrier relates to physical ability, a mind-set, or a combination of the two is not clear.

A reason many women gave for not wanting to self-manage was a lack of confidence[16, 53]. However, despite a lack of confidence undertaking the self-management role, the same women demonstrated high levels of confidence addressing daily challenges with their pessary, such as readjusting it as required[16]. It may also be women feel more able to access support if receiving clinician led care. Conversely, women who commenced pessary self-management reported better access support than those receiving clinician led care[2]. The intimate nature of pessary care is also a consideration, some women reported feeling uncomfortable touching their genitalia[16]. This was also identified by[2] where 11% of women declined self-management due to the nature of pessary care. A similar issue reported by Abdool, Thakar [54] was many women expressing a preference not to handle a pessary. Thirteen percent of women declined self-management, reporting clinicians found removal or insertion difficult and therefore they did not feel able to self-manage the pessary independently[2].

Teaching self-management

At present there are no evidence-based guidelines to specify the requirements of self-management teaching[55]. The identified literature provides some evidence regarding the included constituents of self-management teaching. Where reported, self-management teaching took between 45 minutes and an hour[2, 56]. The level of detail provided about self-management teaching varied greatly. Therefore, it was not always clear whether details of basic instructions were simply a brief description, or an accurate representation of the teaching provided. In all identified studies which provided details about the teaching, women were given instructions on removal and insertion of the pessary[2, 29, 51, 56]. Kearney and Brown [2] specified that women were supervised practicing a pessary change however this was not mentioned elsewhere.

Kearney and Brown [2] state that as well as face to face training, women were provided with written information and access to an online video. Both were designed utilising feedback from pessary users who attended a focus group. Women who received written information were more satisfied, more confident and demonstrated increased knowledge regarding every aspect of pessary care covered by the brochure when compared with women solely given verbal information[48].

For peer review only - http://bmjopen.bmj.com/site/about/guidelines.xhtml

Within the identified literature women were taught to self-manage by specialist nurses[37, 56] or specialist women's health physiotherapist[2]. Kearney and Brown [2] originally intended to have a specialist nurse undertake this role. However, they believe having a physiotherapist who would typically perform a rehabilitative role rather than delivering medical interventions was beneficial to the service evaluation. Only one study evaluated outcomes following self-management teaching. [51] found after only one tutorial, new pessary using women had increased confidence to self-manage their pessary and described it as 'easy'.

Cost benefit

While the primary goal of pessary self-management is an improved experience for women, there is also the potential for cost savings if the number of follow-up appointments required can be reduced both for the woman and the service[29, 51]. While the cost of attending frequent follow-up may be a consideration for women attending appointments anywhere, it is important to clarify the women audited by Goh, Thayalan [51] lived in a 'poor' rural farming area of Uganda, one of the poorest countries in the world[57]. Therefore, the impact of costs to travel to an appointment are likely to be more of a consideration, or deterrent, for women in this audit compared with the general population. Detailed cost analysis of self-management versus clinician-led care suggests potential for annual cost savings of £5,500 for the Trust and £8,400 for commissioners for 50 women[2]. This calculation is based upon 90 minutes of a band six nurse or physiotherapist's time to provide initial self-management teaching and subsequent telephone follow-up. It is important to note, the significant cost savings calculated are based upon clinician-led pessary care being provided by doctors, whereas at most organisations in the UK it is provided by a combination of doctors and nurses[58]. This may affect projected cost savings.

DISCUSSION

Despite a number of factors being associated with likelihood to self-manage[16-24], a causative relationship has not been established with any. As physical ability does not appear to affect willingness to self-manage a pessary[42, 51], further exploration is required to enable us to understand why such a large number of women are unwilling to undertake this role[16, 22, 23, 32, 33, 36, 46-49].

As suggested in the literature, it is unsurprising with increasing practice and confidence, self-management would get easier[51]. It may also be due to pessary related factors such as the position of the pessary changing and becoming harder to reach or more immovable. Alternatively, it might be

For peer review only - http://bmjopen.bmj.com/site/about/guidelines.xhtml

that dynamic characteristics of the woman such as hormonal changes affecting the flexibility or lack of, of the surrounding vaginal tissues, physical flexibility and dexterity fluctuations cause occasional difficulty. Due to the fluctuating nature of self-management ability[42], it is questionable whether a decision can be made upon one assessment. Despite this, there is no robust evidence regarding the skills and ability required to self-manage a pessary, therefore further research in this area is indicated.

The finding of the scoping review demonstrate the lack of evidence to guide decision making regarding pessary self-management. However, there is potential for the weight of evidence to appear greater than it is due to repetition. Ten of the included studies had been solely published in abstract format despite many of them being presented a number of years ago[19, 22, 24, 28, 37, 44, 47, 50, 53, 59]. The decision was made to include these abstracts despite not being published in full to ensure very recent data was included. However, it has highlighted the potential for publication bias in the literature related to self-management.

Despite critical appraisal of evidence not being a traditional component of scoping reviews[7], assessment of the quality of the included papers has enabled us to identify recurring themes in the methodological limitations of the evidence base. This highlights the need for well-designed research studies, particularly randomised controlled trials within this field to improve rigour within the literature. A further limitation is restriction to the four databases searched which may result in failure to identify eligible, relevant publications. The search terms utilised included 'self-management' and 'self-care'. However, it is possible not all centres use these terms to describe teaching women to remove and insert their pessaries. In which case only instances where authors use the terms self-management or self-care or perceive self-management of pessaries as something other than standard care will have been identified.

In conclusion, despite the lack of rigorous evidence, the publications identified in this scoping review suggest pessary self-management offers benefits to women[2, 39] with no increased risk of complications[19, 23, 35, 37, 44]. It is clear some women do not feel willing or able to self-manage their pessary[2, 16, 21-24, 28, 32, 33, 35-37, 41, 44, 46-50]. Therefore, in-depth exploration of women's beliefs and attitude towards pessary self-management is required to further our understanding of the barriers women experience and to determine whether increased support to help women overcome these issues or concerns may address this. Further rigorous evidence is also required to clarify whether or not clinician led follow-up is necessary for self-managing women, or, whether self-managing women could instead request an appointment only if they experience problems or have pessary related concerns. In a cohort of women receiving clinician led care,

For peer review only - http://bmjopen.bmj.com/site/about/guidelines.xhtml

reliance upon patient reported symptoms to identify pessary complications was found to miss 27% of women with an excoriation.[60] However, whether this would be the case in self-managing women who would be removing their pessary more frequently has not been established.

. The findings of the TOPSY study[55] expected to be published in 2022 are anticipated to clarify certain aspects of self-management care. However, further in-depth exploration of factors which affect women's willingness to self-manage their pessary is indicated to ensure the issue is better understood and women are better supported to self-manage their prolapse as those with other conditions have been previously.

For peer review only

For peer review only - http://bmjopen.bmj.com/site/about/guidelines.xhtml

AUTHORS CONTRIBUTIONS

Lucy Dwyer devised the scoping review question, methodology, performed the literature search, data extraction and drafted this manuscript.

Rohna Kearney substantively contributed to the development of the scoping review question, methodology, performed data extraction and critical appraisal of a subsample of the included studies and has revised and approved this manuscript.

Dawn Dowding substantively contributed to the development of the scoping review question, methodology and revised and approved this manuscript.

COMPETING INTERESTS STATEMENT

Lucy Dwyer and Rohna Kearney are co-applicants of the NIHR/HTA funded Treatment of Prolapse with Self-Care Pessary (TOPSY) study.

FUNDING

Lucy Dwyer, Clinical Doctoral Research Fellow, NIHR300519 is funded by Health Education England (HEE) / National Institute for Health Research (NIHR) for this research project. The views expressed in this publication are those of the author(s) and not necessarily those of the NIHR, NHS or the UK Department of Health and Social Care.

DATA SHARING STATEMENT

No additional data available.

For peer review only

For peer review only - http://bmjopen.bmj.com/site/about/guidelines.xhtml

LIST OF TABLES, IMAGES AND SUPPLEMENTARY MATERIAL

Table 1 Summary of studies

Image 1-Inclusion criteria

Image 2-Review flow chart

Image 3- MMAT results

Supplementary material 1-PRISMA checklist

Supplementary material 2-CINAHL search

Supplementary material 3-Embase search

Supplementary material 4-Medline search

Supplementary material 5-PsycINFO search

Supplementary material 6-Data extraction form

For peer review only

REFERENCES

1. Lone, F., R. Thakar, and A.H. Sultan, *One-year prospective comparison of vaginal pessaries and surgery for pelvic organ prolapse using the validated ICIQ-VS and ICIQ-UI (SF) questionnaires.* International Urogynecology Journal, 2015. **26**(9).
2. Kearney, R. and C. Brown, *Self-management of vaginal pessaries for pelvic organ prolapse.* BMJ Quality Improvement Reports, 2014. **3**(1).
3. Basu, M., B. Wise, and J. Duckett, *A qualitative study of women's preferences for treatment of pelvic floor disorders.* BJOG: An International Journal of Obstetrics & Gynaecology, 2011. **118**(3): p. 338-344.
4. Nguyen, J.N. and C.R. Jones, *Pessary treatment of pelvic relaxation: Factors affecting successful fitting and continued use.* Journal of Wound, Ostomy and Continence Nursing, 2005. **32**(4).
5. Bugge, C., et al., *Does self-management of vaginal pessaries improve care for women with pelvic organ prolapse?*, in *The BMJ*. 2021.
6. Munn, Z., et al., *Systematic review or scoping review? Guidance for authors when choosing between a systematic or scoping review approach.* BMC Medical Research Methodology, 2018. **18**(1).
7. Peters, M., et al., *Chapter 11: Scoping Reviews (2020 version).* JBI Reviewer's Manual, 2020.
8. Tricco, A.C., et al., *PRISMA extension for scoping reviews (PRISMA-ScR): Checklist and explanation*, in *Annals of Internal Medicine*. 2018.
9. Hong, Q.N., et al., *The Mixed Methods Appraisal Tool (MMAT) version 2018 for information professionals and researchers.* Education for Information, 2018. **34**(4): p. 285-291.
10. Lough, K., et al., *Shared research priorities for pessary use in women with prolapse: Results from a James Lind Alliance Priority Setting Partnership.* BMJ Open, 2018. **8**(4).
11. Sterne, J., J. Higgins, and B. Reeves, *on behalf of the development group for ACROBAT-NRSI, 2014. A Cochrane Risk Of Bias Assessment Tool: for Non-Randomized Studies of Interventions (ACROBAT-NRSI), Version 1.0. 0*. 2014.
12. Khaja, A. and R.M. Freeman, *How often should shelf/Gellhorn pessaries be changed? A survey of IUGA urogynaecologists.* International Urogynecology Journal, 2014. **25**(7).
13. Bugge, C., S. Hagen, and R. Thakar, *Vaginal pessaries for pelvic organ prolapse and urinary incontinence: A multiprofessional survey of practice.* International Urogynecology Journal and Pelvic Floor Dysfunction, 2013. **24**(6).
14. Cundiff, G., *A survey of pessary use by members of the american urogynecologic society.* Obstetrics & Gynecology, 2000. **95**(6).
15. Althubaiti, A., *Information bias in health research: definition, pitfalls, and adjustment methods.* Journal of multidisciplinary healthcare, 2016. **9**: p. 211-217.
16. Storey, S., et al., *Women's experiences with vaginal pessary use.* Journal of Advanced Nursing, 2009. **65**(11).
17. Tenfelde, S., et al., *Quality of life in women who use pessaries for longer than 12 months.* Female Pelvic Medicine and Reconstructive Surgery, 2015. **21**(3).
18. Meriwether, K.V., et al., *The effect of hydroxyquinoline-based gel on pessary-associated bacterial vaginosis: A multicenter randomized controlled trial.* American Journal of Obstetrics and Gynecology, 2015. **213**(5).
19. Morcuende, I., et al., *Pessary Treatment of Pelvic Organ Prolapse: Self Care vs. Office-based Care.* PFD Week 2018, 2018.
20. Yoshimura, K., et al., *Changes of intravaginal microbiota and inflammation after self-replacement ring pessary therapy compared to continuous ring pessary usage for pelvic organ prolapse.* Journal of Obstetrics and Gynaecology Research, 2020. **46**(6).
21. Thys, S.D., et al., *Effect of pessary cleaning and optimal time interval for follow-up: a prospective cohort study.* International Urogynecology Journal, 2020. **31**(8).

For peer review only - http://bmjopen.bmj.com/site/about/guidelines.xhtml

22. Chen, Y.B., et al., *The pessary fits, now what? clinical factors associated with pessary self-management Vs. Clinic management.* Female Pelvic Medicine and Reconstructive Surgery, 2020. **26**(10 SUPPL 1): p. S132-S133.
23. Holubyeva, A., et al., *Rates of Pessary Self-Care and the Characteristics of Patients Who Perform it.* Female pelvic medicine & reconstructive surgery, 2021. **27**(3).
24. Ramsay, S., F. Bouchard, and L.M. Tu, *Long term outcomes of pessary use in women with pelvic organ prolapse.* Neurourology and Urodynamics, 2011. **30**(6).
25. Dwyer, L. and R. Kearney, *Conservative management of pelvic organ prolapse.* Obstetrics, Gynaecology & Reproductive Medicine, 2018. **28**(1): p. 15-21.
26. Rantell, A., *Vaginal Pessaries for Pelvic Organ Prolapse and Their Impact on Sexual Function.* Sex Med Rev, 2019. **7**(4): p. 597-603.
27. Meriwether, K.V., et al., *Sexual Function and Pessary Management among Women Using a Pessary for Pelvic Floor Disorders.* The Journal of Sexual Medicine, 2015. **12**(12).
28. Pizarro-Berdichevsky, J., et al., *Pessary use in patients with symptomatic pelvic organ prolapse-12 month prospective study of factors predictive of subjective improvement.* International Urogynecology Journal and Pelvic Floor Dysfunction, 2016. **27**(1).
29. Manonai, J., S. Sarit-Apirak, and U. Udomsubpayakul, *Vaginal ring pessary use for pelvic organ prolapse: Continuation rates and predictors of continued use.* Menopause, 2019. **26**(6).
30. Ma, C., et al., *Vaginal pessary treatment in women with symptomatic pelvic organ prolapse: a long-term prospective study.* Menopause (New York, N.Y.), 2021. **28**(5).
31. Clemons, J.L., et al., *Patient characteristics that are associated with continued pessary use versus surgery after 1 year.* American Journal of Obstetrics and Gynecology, 2004. **191**(1).
32. Chan, M.C., et al., *What Are the Clinical Factors That Are Predictive of Persistent Pessary Use at 12 Months?* Journal of Obstetrics and Gynaecology Canada, 2019. **41**(9).
33. Wu, V., et al., *A simplified protocol for pessary management.* Obstetrics and Gynecology, 1997. **90**(6).
34. Bai, S.W., et al., *Survey of the characteristics and satisfaction degree of the patients using a pessary.* International Urogynecology Journal, 2005. **16**(3).
35. Manchana, T., *Ring pessary for all pelvic organ prolapse.* Archives of Gynecology and Obstetrics, 2011. **284**(2).
36. Clemons, J.L., et al., *Patient satisfaction and changes in prolapse and urinary symptoms in women who were fitted successfully with a pessary for pelvic organ prolapse.* American Journal of Obstetrics and Gynecology, 2004. **190**(4).
37. Daneel, L., N.I. te West, and K.H. Moore, *Does monthly self-removal of vaginal ring pessaries for stress incontinence/ prolapse reduce complication rates? A 5 year audit.* Neurourology and Urodynamics, 2016.
38. Chien, C.W., et al., *Long-term outcomes of self-management gellhorn pessary for symptomatic pelvic organ prolapse.* Female Pelvic Medicine and Reconstructive Surgery, 2020. **26**(11).
39. Hanson, L.A.M., et al., *Vaginal pessaries in managing women with pelvic organ prolapse and urinary incontinence: Patient characteristics and factors contributing to success.* International Urogynecology Journal, 2006. **17**(2).
40. Cooper-Surgical. *Cube and Tandem Cube Pessary Instructions for Use.* 2017; Available from: www.coopersurgical.com/product-resources/7c3c5e98-68d5-4d44-b2fc-3d6bae7d2fad_Cube-and-Tandem-Cube-Pessary-Instructions-for-Use.pdf
41. Nemeth, Z., S. Nagy, and J. Ott, *The cube pessary: An underestimated treatment option for pelvic organ prolapse? Subjective 1-year outcomes.* International Urogynecology Journal and Pelvic Floor Dysfunction, 2013. **24**(10).
42. Sulak, P.J., T.J. Kuehl, and B.L. Shull, *Vaginal pessaries and their use in pelvic relaxation.* Journal of Reproductive Medicine for the Obstetrician and Gynecologist, 1993. **38**(12).

For peer review only - http://bmjopen.bmj.com/site/about/guidelines.xhtml

43. Gorti, M., G. Hudelist, and A. Simons, *Evaluation of vaginal pessary management: A UK-based survey.* Journal of Obstetrics and Gynaecology, 2009. **29**(2).
44. Lammers, K., et al., *Does monthly self-management of vaginal ring pessaries reduce complication rates?* International Urogynecology Journal, 2019. **30**(1 Supplement): p. S271-S272.
45. Fregosi, N.J., et al. *Changes in the Vaginal Microenvironment as Related to Frequency of Pessary Removal*. in *Female Pelvic Medicine and Reconstructive Surgery*. 2018.
46. Clemons, J.L., et al., *Risk factors associated with an unsuccessful pessary fitting trial in women with pelvic organ prolapse.* American Journal of Obstetrics and Gynecology, 2004. **190**(2).
47. Jacobs, S. and E. Banks, *Pilot study: Pessary use in bronx women. Would increasing the practice of self-changing pessaries have potential to positively impact body image?* Journal of Pelvic Medicine and Surgery, 2010. **16**(5 SUPPL. 2): p. S143-S144.
48. Murray, C., E. Thomas, and W. Pollock, *Vaginal pessaries: can an educational brochure help patients to better understand their care?* Journal of Clinical Nursing, 2017. **26**(1-2).
49. Tam, M.S., et al., *The effect of time interval of vaginal ring pessary replacement for pelvic organ prolapse on complications and patient satisfaction: A randomised controlled trial.* Maturitas, 2019. **128**.
50. Hooper, G., et al., *Cube pessary use and quality of life in women with pelvic organ prolapse.* Female Pelvic Medicine and Reconstructive Surgery, 2018. **24**(5).
51. Goh, J.T.W., K. Thayalan, and H.G. Krause, *An audit of vaginal support pessaries for pelvic organ prolapse in western Uganda.* Australian and New Zealand Continence Journal, 2020. **26**(2).
52. Sarma, S., T. Ying, and K.H. Moore, *Long-term vaginal ring pessary use: Discontinuation rates and adverse events.* BJOG: An International Journal of Obstetrics and Gynaecology, 2009. **116**(13).
53. Ibrahim, S., et al., *Evaluation of pessary service for women with pelvic organ prolapse (POP).* International Journal of Gynecology and Obstetrics, 2015. **131**(SUPPL. 5): p. E164.
54. Abdool, Z., et al., *Prospective evaluation of outcome of vaginal pessaries versus surgery in women with symptomatic pelvic organ prolapse.* International Urogynecology Journal, 2011. **22**(3).
55. Hagen, S., et al., *Clinical and cost-effectiveness of vaginal pessary self-management compared to clinic-based care for pelvic organ prolapse: protocol for the TOPSY randomised controlled trial.* Trials, 2020. **21**(1): p. 1-13.
56. Kuhn, A., et al., *Sexual and organ function in patients with symptomatic prolapse: are pessaries helpful?* Fertility and Sterility, 2009. **91**(5).
57. The World Bank. *The Uganda Poverty Assessment Report 2016*. 2016; Available from: https://thedocs.worldbank.org/en/doc/381951474255092375-0010022016/original/UgandaPovertyAssessmentReport2016.pdf.
58. Dwyer, L., E. Stewart, and A. Rajai, *A service evaluation to determine where and who delivers pessary care in the UK.* Int Urogynecol J, 2021. **32**(4): p. 1001-1006.
59. Chen, Y.B., et al., *Pessary self-management vs. clinic management: Is one more likely to result in surgery?* Female Pelvic Medicine and Reconstructive Surgery, 2020. **26**(10 SUPPL 1): p. S151-S152.
60. Davis, C., et al., *IUGA 43rd Annual Meeting – Vienna, Austria, June 27-30, 2018.* International Urogynecology Journal, 2018. **29**(1): p. 1-272.

For peer review only - http://bmjopen.bmj.com/site/about/guidelines.xhtml

Image 1-Inclusion and Exclusion criteria

Inclusion criteria

- Original research
- Pessary for POP
- Published in English language
- Focuses on self-management of pessary for POP

Exclusion criteria

- Not relevant to subject area
- Not published in English language
- Not original research including case reports

For peer review only

Image Two-Flow chart

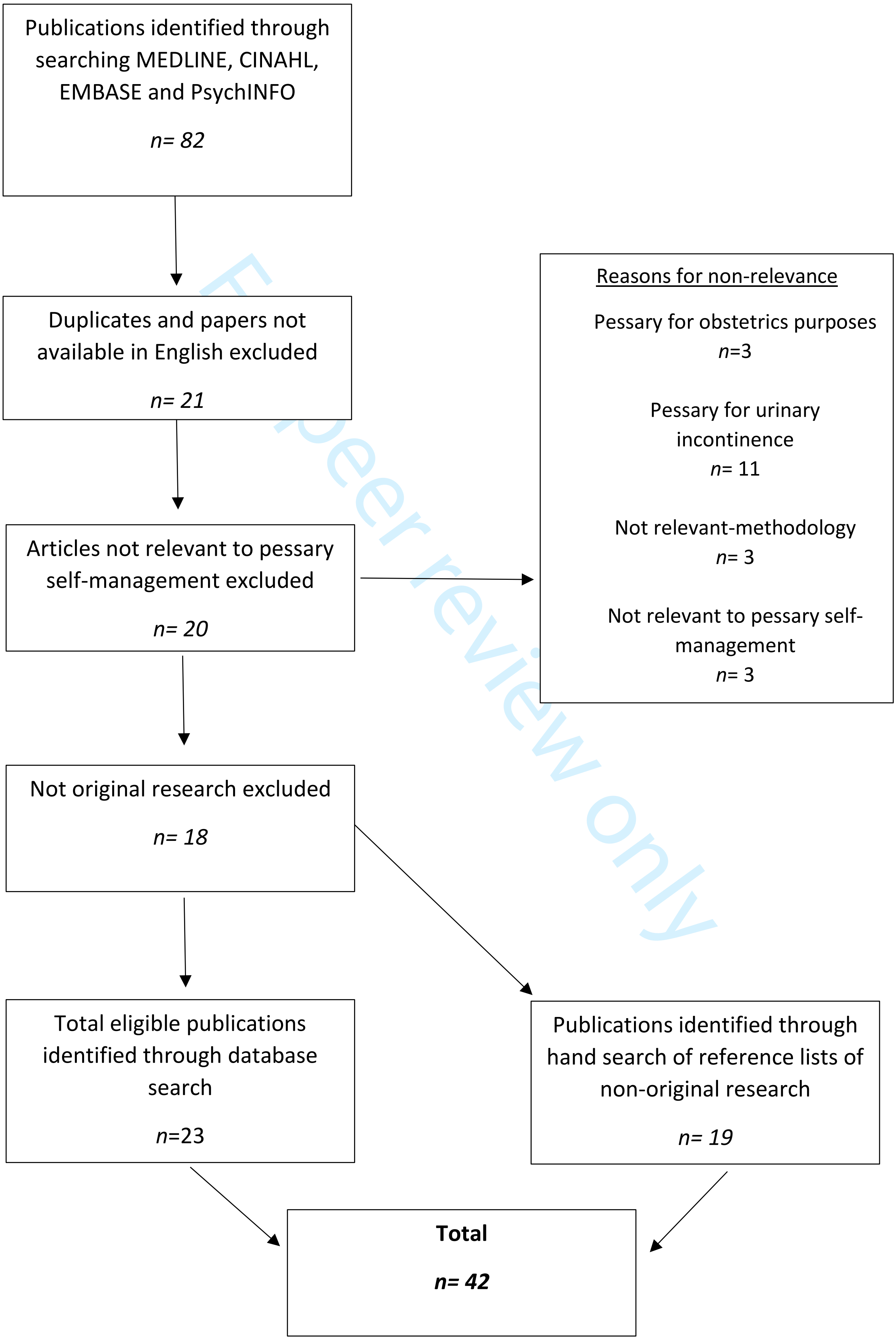

For peer review only

Figure One-MMAT

	1. Qualitative studies					2. Quantitative randomised controlled trial					3. Quantitative non-randomized studies					4. Quantitative descriptive studies				
	1.1-Appropriate approach	1.2-Adequate data collection methods	1.3 Are findings adequately derived?	1.4- Interpretation sufficiently substantiated	1.5-Coherence	2.1-Appropriate randomisation	2.2- Comparable groups at baseline	2.3- Outcome data completeness	2.4-Blinging	2.5-Adherence to randomisation	3.1-Representativeness	3.2-Measurements appropriate	3.3-Outcome data completeness	3.4-Confounders accounted for	3.5-Exposure as intended	4.1-Relevant sampling strategy	4.2- Representativeness	4.3- Measurements appropriate	4.4-Nonresponse bias	4.5-Appropriate statistical analysis
Abdool et al, 2011											1	1	1	1	1					
Bai et al, 2005																1	0	0	1	1
Brown et al, 2016																1	0	1	0	1
Bugge et al, 2013																1	0	1	0	1
Chan et al, 2019											1	1	0	1	1					
*Chen et al, 2020a and Chen et al, 2020b**											1	0	1	1	1					
Chien et al, 2020											0	0	0	0	1					
Clemons et al. 2004a, Clemons et al. 2004b and Clemons et al. 2004c**											1	1	1	1	1					
Cundiff et al, 2000																1	1	1	0	1
*Daneel et al, 2016																1	1	0	1	0
Goh et al, 2020																1	0	1	1	1
Hanson et al, 2006											1	0	1	0	1					
Holubyeva et al, 2021											0	0	1	0	1					
*Hooper et al, 2018											1	1	1	0	1					
*Ibrahim et al, 2015																1	1	1	0	1
*Jacobs and Banks, 2010																1	1	1	0	1
Kearney and Brown, 2014											1	0	1	0	1					
Khaja and Freeman, 2014																1	0	1	0	1
*Lammers et al, 2019											1	0	1	0	1					
Kuhn et al, 2008																1	1	1	1	1
Ma et al, 2001											1	1	1	1	1					
Manchana, 2011											1	1	1	0	1					
Manonai et al, 2018											1	0	0	0	1					
Meriwether et al, 2015a, Meriwether et al, 2015b and Fregosi et al, 2018**						1	1	0	1	1										
*Morcuende et al, 2018											1	0	1	0	0					
Murray et al, 2017											1	1	1	0	1					
Nemeth et al, 2013																1	1	1	1	1
*Pizarro-Berdichevsky et al, 2016											1	1	1	1	1					
Ramsay et al, 2011											1	0	0	1	1					
Sarma et al, 2009																1	1	0	0	1
Storey et al, 2009	1	1	1	1	1															
Sulak et al, 1993																0	0	0	1	1
Tam et al, 2019						1	1	1	1	1										
Thys et al, 2020											1	1	0	0	1					
Tenfelde et al, 2015																1	1	1	1	1
Wu et al, 1997																1	1	1	1	1
Yoshimura et al, 2020											1	1	1	0	0					

* Abstract

** Clustered studies where secondary analysis of the same sample of women was performed

http://mixedmethodsappraisaltoolpublic.pbworks.com/w/file/fetch/140056890/Reporting%20the%20results%20of%20the%20MMAT.pdf

For peer review only

Supplementary material One

PRISMA 2020 Checklist

Section and Topic	Item #	Checklist item	Location where item is reported
**TITLE**			
Title	1	Identify the report as a systematic review.	Page 1
**ABSTRACT**			
Abstract	2	See the PRISMA 2020 for Abstracts checklist.	Page 2
**INTRODUCTION**			
Rationale	3	Describe the rationale for the review in the context of existing knowledge.	Page 4
Objectives	4	Provide an explicit statement of the objective(s) or question(s) the review addresses.	Page 5
**METHODS**			
Eligibility criteria	5	Specify the inclusion and exclusion criteria for the review and how studies were grouped for the syntheses.	Page 8
Information sources	6	Specify all databases, registers, websites, organisations, reference lists and other sources searched or consulted to identify studies. Specify the date when each source was last searched or consulted.	Page 7
Search strategy	7	Present the full search strategies for all databases, registers and websites, including any filters and limits used.	To be included with results
Selection process	8	Specify the methods used to decide whether a study met the inclusion criteria of the review, including how many reviewers screened each record and each report retrieved, whether they worked independently, and if applicable, details of automation tools used in the process.	Page 12
Data collection process	9	Specify the methods used to collect data from reports, including how many reviewers collected data from each report, whether they worked independently, any processes for obtaining or confirming data from study investigators, and if applicable, details of automation tools used in the process.	Page 12
Data items	10a	List and define all outcomes for which data were sought. Specify whether all results that were compatible with each outcome domain in each study were sought (e.g. for all measures, time points, analyses), and if not, the methods used to decide which results to collect.	Page 12
	10b	List and define all other variables for which data were sought (e.g. participant and intervention characteristics, funding sources). Describe any assumptions made about any missing or unclear information.	Page 12
Study risk of bias assessment	11	Specify the methods used to assess risk of bias in the included studies, including details of the tool(s) used, how many reviewers assessed each study and whether they worked independently, and if applicable, details of automation tools used in the process.	Page 13-14
Effect measures	12	Specify for each outcome the effect measure(s) (e.g. risk ratio, mean difference) used in the synthesis or presentation of results.	Page 13-14
Synthesis methods	13a	Describe the processes used to decide which studies were eligible for each synthesis (e.g. tabulating the study intervention characteristics and comparing against the planned groups for each synthesis (item #5)).	Page 15
	13b	Describe any methods required to prepare the data for presentation or synthesis, such as handling of missing summary statistics, or data conversions.	Page 15
	13c	Describe any methods used to tabulate or visually display results of individual studies and s 4yntheses.	Page 15
	13d	Describe any methods used to synthesize results and provide a rationale for the choice(s). If meta-analysis was performed, describe the model(s), method(s) to identify the presence and extent of statistical heterogeneity, and software package(s) used.	Page 14-15

For peer review only - http://bmjopen.bmj.com/site/about/guidelines.xhtml

Supplementary material One

PRISMA 2020 Checklist

Section and Topic	Item #	Checklist item	Location where item is reported
	13e	Describe any methods used to explore possible causes of heterogeneity among study results (e.g. subgroup analysis, meta-regression).	Page 15
	13f	Describe any sensitivity analyses conducted to assess robustness of the synthesized results.	Page 15
Reporting bias assessment	14	Describe any methods used to assess risk of bias due to missing results in a synthesis (arising from reporting biases).	Page 15
Certainty assessment	15	Describe any methods used to assess certainty (or confidence) in the body of evidence for an outcome.	Page 15
**RESULTS**			
Study selection	16a	Describe the results of the search and selection process, from the number of records identified in the search to the number of studies included in the review, ideally using a flow diagram.	To be included with results
	16b	Cite studies that might appear to meet the inclusion criteria, but which were excluded, and explain why they were excluded.	To be included with results
Study characteristics	17	Cite each included study and present its characteristics.	To be included with results
Risk of bias in studies	18	Present assessments of risk of bias for each included study.	To be included with results
Results of individual studies	19	For all outcomes, present, for each study: (a) summary statistics for each group (where appropriate) and (b) an effect estimate and its precision (e.g. confidence/credible interval), ideally using structured tables or plots.	To be included with results
Results of syntheses	20a	For each synthesis, briefly summarise the characteristics and risk of bias among contributing studies.	To be included with results
	20b	Present results of all statistical syntheses conducted. If meta-analysis was done, present for each the summary estimate and its precision (e.g. confidence/credible interval) and measures of statistical heterogeneity. If comparing groups, describe the direction of the effect.	To be included with results
	20c	Present results of all investigations of possible causes of heterogeneity among study results.	To be included with results
	20d	Present results of all sensitivity analyses conducted to assess the robustness of the synthesized results.	To be included with results

For peer review only

Supplementary material One

PRISMA 2020 Checklist

Section and Topic	Item #	Checklist item	Location where item is reported
Reporting biases	21	Present assessments of risk of bias due to missing results (arising from reporting biases) for each synthesis assessed.	To be included with results
Certainty of evidence	22	Present assessments of certainty (or confidence) in the body of evidence for each outcome assessed.	To be included with results
**DISCUSSION**			
Discussion	23a	Provide a general interpretation of the results in the context of other evidence.	To be included with results
	23b	Discuss any limitations of the evidence included in the review.	To be included with results
	23c	Discuss any limitations of the review processes used.	To be included with results
	23d	Discuss implications of the results for practice, policy, and future research.	To be included with results
**OTHER INFORMATION**			
Registration and protocol	24a	Provide registration information for the review, including register name and registration number, or state that the review was not registered.	Page 6
	24b	Indicate where the review protocol can be accessed, or state that a protocol was not prepared.	This publication
	24c	Describe and explain any amendments to information provided at registration or in the protocol.	n/a
Support	25	Describe sources of financial or non-financial support for the review, and the role of the funders or sponsors in the review.	Page 17
Competing interests	26	Declare any competing interests of review authors.	Page 17
Availability of data, code and other materials	27	Report which of the following are publicly available and where they can be found: template data collection forms; data extracted from included studies; data used for all analyses; analytic code; any other materials used in the review.	To be included with results

From: Page MJ, McKenzie JE, Bossuyt PM, Boutron I, Hoffmann TC, Mulrow CD, et al. The PRISMA 2020 statement: an updated guideline for reporting systematic reviews. BMJ 2021;372:n71. doi: 10.1136/bmj.n71

For more information, visit: http://www.prisma-statement.org/

For peer review only

EBSCOhost

Supplementary material 2

Monday, October 18, 2021 4:07:34 PM

#	Query	Limiters/Expanders	Last Run Via	Results
S1	pessary AND (self management or self care)	Expanders - Apply equivalent subjects<br>Search modes - Boolean/Phrase	Interface - EBSCOhost Research Databases<br>Search Screen - Advanced Search<br>Database - CINAHL Plus	23

508x255mm (96 x 96 DPI)

<u>Supplementary material 3</u>

Embase <1974 to 2021 May 07>

1	self care/	65562
2	vagina pessary/	3004
3	1 and 2	44

For peer review only

Supplementary material 4

Ovid MEDLINE(R) <1946 to May Week 1 2021>

1	Pessaries/	1578
2	Self Care/ or Self-Management/	37957
3	1 and 2	16

For peer review only

Supplementary material 5

APA PsycInfo <1806 to May Week 4 2021>

1	exp Self-Management/	7368
2	exp Self-Care/	2977
3	1 or 2	10087
4	pessary.mp.	9
5	pessaries.mp.	14
6	4 or 5	17
7	3 and 6	0

For peer review only

Supplementary Material 6-Data Extraction Form

Database	Author and date of publication	Title	Population studied	Context	Methods	Type(s) of pessary	Stage of prolapse	Age of participants	Follow-up	Key findings related to pessary self-management
Medline	Yoshimura et al, 2020	Changes of intravaginal microbiota and inflammation after self-replacement ring pessary therapy compared to continuous ring pessary usage for pelvic organ prolapse.	50 pessary using women	Attending clinic in University hospital in Japan	Laboratory study	Wallace Ring Pessary	Mean 3	Mean 58 years	At 1 month	Reduction in PV discharge with SM, Younger patients tended to learn SM, Abnormal vaginal microbiota as frequently seen in SM group.

Medline	Murray et al, 2016	Vaginal pessaries: can an educational brochure help patients to better understand their care?	60 women with stage 2 or 3 symptomatic prolapse	Attending pessary clinic in hospital Australia	Prospective study with pretest-posttest	Not reported	Not reported	Mean 69 years	At 1 week and 3 months	85% read the SM brochure at 1/52 and 97% at 2/52, Increased satisfaction with SM information given with patient information brochure than with verbally alone, Increased confidence SM with written information, Brochure= increased happiness with information given, increased confidence in SM, increased knowledge re: POP, Increased knowledge re: what a pessary does, increased knowledge re: what to do if a pessary falls out, increased knowledge re: when to use

										oestrogen, increased knowledge regarding what to do if experiencing discharge, vaginal bleeding, a UTI or urinary leakage.
Medline	Tenfelde et al, 2015	Quality of life in women who use pessaries for longer than 12 months.	56 pessary using women	Attending tertiary clinic in USA	Observational cross-sectional study	Not reported	Not reported	Mean 74 years	No follow-up	Most SM women removed pessary daily (40%) or a few times per week (40%). 20% Removed pessary monthly or greater. 44% of women self-managing pessary.
Medline	Manchana, 2011	Ring pessary for all pelvic organ prolapse.	100 women with symptomatic POP	Attending outpatient gynaecology clinic in Thailand	Retrospective case note review	Ring pessary	Mean 3	Median 71 years	Median 13 months	Only 25 women (40%) could SM by themselves or with caregivers assistance. 4 of 25 SM women (16%) and 23 of 37 non SM women (62%) had adverse events e.g. discharge. Self-

For peer review only

										care protocol given as a reason for pessary discontinuation. The ring pessary is easy to teach patient to SM.
Embase	Ma et al, 2021	Vaginal pessary treatment in women with symptomatic pelvic organ prolapse: a long-term prospective study.	312 women with symptomatic prolapse receiving pessary management	Attending tertiary referral centre in China	Prospective observational study	Ring or gell-horn pessary	Mean 3	Mean 69 years	At 5 years	Women were advised to perform weekly self-care. Inability to SM a potential factor in discontinuing pessary management.
Embase	*Chen et al, 2020a	The pessary fits, now what? clinical factors associated with pessary self-management Vs.	218 long term pessary users (excluded those using as interim before surgery or did not complete trial)	Tertiary urogynaecology clinic USA	Retrospective notes review	Not reported	Not reported	Median age 66 years	Not reported	Of 218 women, 159 were self-managing (73%). Increased age, increased BMI, increased prolapse stage, type of pessary and raised POPQ score for Ba, Bp, C and GH increased the

For peer review only

		Clinic management.								odds of office management.
Embase	Holubyeva, 2021	Rates of pessary self-care and the characteristics of patients who perform it.	Notes of 580 women receiving pessary care	Tertiary care centre in USA	Retrospective notes review	Cube, Donut, Gellhorn, Incontinence dish, Incontinence ring, ring, Ring with support and other	Unable to determine due to grouping of prolapse stages	Mean 72 years	No follow-up	31% (174/563) chose to SM. Patients performing SM were seen less frequently (4-6 monthly) whereas non-SM women were seen 1-3 monthly. Less SM experienced PVB (16% vs 26%) and vaginal erosions (10% vs 24%). Patients in SM group significantly younger. Post-menopausal women more likely to want office care. Women with lower stage prolapse (1-2) more likely to SM. SM women more likely to be fitted with a ring

For peer review only

										pessary or ring with support whereas office-based care women more likely to be fitted with a gell-horn or ring with support. Women who opted not to SM had a slightly higher BMI, increased rates of arthritis and cognitive impairment, however none of this was statistically significant.
Embase	*Chen et al, 2020b	Pessary self-management vs. clinic management: Is one more likely to result in surgery?	218 long term pessary users (excluded those using as interim before surgery or did not complete trial)	Tertiary urogynaecology clinic USA	Retrospective notes review	Not reported	Not reported	Median age 66 years	Not reported	Of 159 SM women, 30 (19%) had surgery and of 59 office managed women, 20 (35% had surgery.

For peer review only

Embase	Chien et al, 2020	Long-term outcomes of self-management gellhorn pessary for symptomatic pelvic organ prolapse	Notes of 93 women using gell-horn pessary	Tertiary urogynecology unit in Taiwan	Retrospective notes review	Gellhorn pessary	Mean 3	Mean 68 years	Median 50 months	Of 421 women referred for gell-horn SM teaching, 147 elected to proceed-note to self, unsure whether that was just SM or gell-horn itself. Patients taught how to deal with and maintain the gell-horn pessary at home. Of 421 women referred for gell-horn SM teaching, 147 elected to proceed-note to self, unsure whether that was just SM or gell-horn itself. At study end point, 48% had used gell-horn continuously with a mean duration of 50 months. 53 (57%) of women reported AEs. 37

For peer review only

										(40%) had vaginal pain or discomfort with gell-horn in situ or during removal or insertion. Elected for surgery, inconvenience, discomfort while pessary in situ or being removed or inserted or resolution of pessary symptoms. Told to wash and reinsert when bathing.
Embase	*Lammers et al, 2019	Does monthly self-management of vaginal ring pessaries reduce complication rates?	Notes of 75 women taught to SM their pessary	Tertiary referral urogynaecology unit in Australia	Retrospective notes review	PVC ring, continence dish, schaatz introl and gellhorn pessary	Not reported	Median 62 years	Median 23 months	5 women had vaginal erosions (7%) and 3 women had minor complications (4%) which resolved with temporary pessary removal. Overall complication rate was 12%

For peer review only

										which compares with 56% of non SM women (complications classed as PVB or discharge. 5 women had vaginal erosions (7%) and 3 women had minor complications (4%) which resolved with temporary pessary removal. Overall complication rate was 12% which compares with 56% of non SM women (complications classed as PVB or discharge). 4 (7%) proceeded to surgery.
Embase	*Morcuende et al, 2018	Pessary treatment of pelvic organ prolapse: Self care	275 women with POP who had pessary management for	Hospital in America	Retrospective records review	Gellhorn and cube pessaries specifically reported, other types	Not reported	Not reported	Median 17 months	SM women seen yearly or PRN compared with 2-3 monthly. SM group were younger, more

For peer review only

		vs. office-based care.	longer than 3 months			included but no details of type				sexually active, had fewer comorbidities and fewer stage 3 and 4 prolapse. Women in the office care group were more likely to have a space occupying pessary or have required a change in size or type of pessary before. Office care was associated with increased rate of erosions in each group despite adjusting for age, comorbidities, POP stage and pessary type. Non SM women more likely to have vaginal erosions.
Embase	*Hooper et al, 2018	Cube pessary use and quality of	25 women with symptomatic POP	Hospital in America	Prospective, observational	Cube pessary	Not reported	Age range 37-92	Not reported	12 or 25 women were able to self-manage cube pessary.

For peer review only

		life in women with pelvic organ prolapse	fitted with a pessary		multi-site study					Cube pessary improved QOL and vaginal symptoms
Embase	Chan et al, 2019	What are the clinical factors that are predictive of persistent pessary use at 12 months?	152 New pessary fittings in patients	Tertiary care urogyna ecology clinic in Canada	Retrospec tive records review	Ring, schatz, incontinence dish, gellhorn	Not reported	Mean 61 years	12 months	Mode of care: self-care (65% n=98) nurse-care (26% n=39). Protocol advised women not to leave the pessary in situ for longer than 3/12. Many women performed self-removal and cleaning on a daily or weekly basis. Nurse vs Self-care not statistically significant to continuation of pessary use at 12/12.
Embase	*Pizarro-Berdichevs ky et al, 2016	Pessary use in patients with symptom	133 women who have used a pessary for a year or	Hospital in Chile	Prospecti ve questionn aire/note s study	Ring, Gellhorn, Donut and other	81% were stage 3 or 4	Mean 65 years	12 months	68% of patients were able to SM. Inability to SM linked with drop out.

		atic pelvic organ prolapse-12 month prospective study of factors predictive of subjective improvement	longer POP stage 2+							
Embase	*Ibrahim et al, 2015	Evaluation of pessary service for women with pelvic organ prolapse (POP)	104 women attending pessary clinic	Different hospitals in Scotland	Questionnaire study	Ring or other	Not reported	Not reported	Not reported	9% of women attending the pessary clinic would consider SM. A common reason for not wanting to SM was lack of confidence.
Embase	Khaja and Freeman, 2014	How often should shelf/Gellhorn pessaries be changed? A survey of IUGA	322 Members of IUGA or BSUG	International	Electronic questionnaire study	Not applicable	Not applicable	Not applicable	Not applicable	Self-care usually acceptable with ring pessary. 86% (n=277) do not believe shelf or gell-horn pessaries are suitable for self care.

For peer review only

		urogynaecologists								

For peer review only

Embase	Kearney and Brown, 2014	Self-management of vaginal pessaries for pelvic organ prolapse	134 pessary using women	Tertiary urogynaecology clinic in Cambridge	Quality improvement and evaluation	Ring and sieve pessary	Not reported	29-92 years	6 months	73% of women who agreed to commence SM were SM at 6/12. No difference in ability to SM depending on size of pessary. SM women had increased level of comfort with pessary changes. SM women reported increased convenience. SM women reported increased access to help. SM women reported increased feeling of support. SM women had increased level of comfort with pessary changes. 1 complication of increased discharge advised to remove the pessary less frequently.

For peer review only

										Reasons for pessary removal n=45<br>• 6/12 change (36% n=16)<br>• To clean (31% n=14)<br>• To aid defecation (11% n=5)<br>• Discomfort (n=10 22%)<br>• To have sex (n=6 13%)<br>• Smear or other procedure (n=2 4%)<br>• Holiday (n=1 2%)<br><br>47 women declined SM due to:<br>• Physical barriers (33% and n=15)<br>• Prefers Dr led care (17% n=8)<br>• Clinician found pessary removal/insertion difficult (13% n=6)

For peer review only

										• Nature of intervention (11% n=5)<br>• Too painful (9% n=4)<br>• Emotional barrier (9% n=4)<br>• Feels too old (6% n=3)<br>• Poor cognitive ability (6% n=3)<br>• Travel arrangements to return for teach SM 4% n=2)<br>• Personal circumstance (4% n=2)<br><br>24 women (27%) stopped SM by 6/12 due to:<br>• Manual dexterity (n=9 38%)<br>• PVB (n=1 4%) and therefore Dr led care<br>• Switched to shelf pessary (n=1 4%) and therefore Dr led care

For peer review only

										• Discontinued pessary management (n=11 46%) • Opted for Surgical management (n=2 8%). Frequency of pessary removal and insertion ranged from twice a week to once every 6 months. More women were able/willing to SM with a ring than sieve pessary. 97% of SM women plan to use pessary long term compared with 70% in Dr care group. At 6/12 of SM 17 of 25 women (68%) were no longer considering surgery. Cost saving for Trust and

For peer review only

										commissioners. The teaching package was delivered by a specialist women's health physiotherapist. Recruiting a physiotherapist to this project was not something that we had originally considered as we had initially seen this as a specialist nursing role. However, we believe that by having a physiotherapist (a role which traditionally focuses on rehabilitation and not medical intervention) at the heart of the service, we have been able to develop something which is truly focussed

For peer review only

										on empowering the patient to manage their own condition away from a healthcare setting. During a single 45-minute appointment, informed consent was obtained and the woman was shown and supervised in pessary change. This face to face training was supplemented with written information and an online teaching video. Feedback from the focus group on the information leaflets was used to inform the structure of the teaching video, with increased emphasis placed

For peer review only

										on simple diagrammatic presentation of prolapse and pessary in situ

For peer review only

base	Nemeth et al, 2013.	The cube pessary: An underestimated treatment option for pelvic organ prolapse? Subjective 1-year outcomes.	87 women with stage 2 or 3 prolapse	Urogynaecology clinic in teaching hospital Hungary	Prospective case review	Cube pessary	Median 3	Median 60 years	12 months	85.5% of women rated SM with a cube pessary as very easy or easy
Embase	*Jacobs and Banks, 2010	Pilot study: Pessary use in Bronx women. Would increasing the practice of self-changing pessaries have potential to positively impact	25 women attending a pessary follow-up clinic	Urogynaecology clinic in hospital USA	Questionnaire study	Not reported	Not reported	Mean 78 years	No follow-up	Despite a high level of function in everyday life, only 50% of women felt capable to SM. 1 of 25 women SM. Only a further 2 felt able to attempt. Only 4 would be willing to try. 12 replied that they would like to change their pessary but felt they couldn't. Body image and function scores

		body image?								equivalent to non pessary users therefore these factors do not appear to link with willingness to SM.
CINAHL	Goh et al, 2020	An audit of vaginal support pessaries for pelvic organ prolapse in western Uganda	93 women with stage 3 or 4 prolapse	Fistula/ prolapse camp in Western Uganda	Audit	R-POP, S-POP and C-POP pessaries	Median 4	Mean 57 years	24 month follow-up	All women (93) taught to SM. 65 trialled pessary for 2 days successfully and were able to SM. Taught to remove once weekly, wash, dry and reinsert. 54 (83%) described SM as easy and had increased confidence in being able to do this after one tutorial.
CINAHL	*Daneel et al, 2016	Does monthly self-removal of vaginal ring pessaries	74 women trained in self-management of ring for POP/SUI	Tertiary urogynaecology clinic in Australia	Audit	Portex ring, Schaatz, Gellhorn, Introl pessary and	Not reported	Median 62 years	Median 23 months	At FU, 66% of women still SM. Complication rate for long term SM women was 10%. Lower complication

For peer review only

		for stress urinary incontinence/ prolapse reduce complication rates? A 5 year audit.				Continence Dish				rate for SM women compared with non-SM women in other studies. Patient wanted surgery, unable to SM due to barriers such as obesity, arthritic fingers) and pessary related complications. Taught to SM by CNS. Women SM rings, Shaatz and gell-horns. Annual FU if SM
CINAHL	Manonai et al, 2018	Vaginal ring pessary use for pelvic organ prolapse: continuation rates and predictors of continued use.	289 women with symptomatic stage 2, 3 and 4 POP	Tertiary centre Thailand	Retrospective chart review	Ring with support	Median 3	Median 71 years	36 months	Self-management protocol included general education re: pessary, self-care coaching re insertion, removal and cleaning, written information and a hotline to call with any emergencies. Self-care of a

For peer review only

										pessary was the only significant factor identified that influenced the continuation rate with pessary management at 3 years. Women who did not require assistance for pessary changes from a healthcare professional or family member were significantly more likely to continue with pessary use.
CINAHL	Storey et al, 2009	Women's experiences with vaginal pessary use.	11 post-menopausal women	Urogynaecology clinic in Canada	Narrative inquiry was used to conduct face-to-face semi-structured interviews.	Not reported	Not reported	All over 65 years	Not applicable	Of 11, only 3 opted to SM. Women referred to being older as a reason not to want to SM. The women who SM reported that they chose it due to confidence in their ability to do so and desire for

For peer review only

										flexibility in when and how they used their pessary. Reasons for not wanting to SM were discomfort ‘touching themselves’, lack of confidence with removal and insertion. Despite this, the women who declined SM showed high levels of confidence in dealing with daily challenges such as adjusting their pessary after opening their bowels. Women described clinic visits as psychological and emotional support as well as physical and pessary care and viewed them as

For peer review only

										social outings. Possible reason not to want to SM.
Handsearch	Meriwether et al, 2015a	The effect of hydroxyquinoline-based gel on pessary associated BV	184 women attending pessary clinic	2 tertiary care centres in USA	Multicentre RCT	Silicone pessary-type not specified	Not reported	Mean 56 years	3 months	Frequency of pessary use or removal did not affect the groups in terms of BV or vaginal symptoms at 2 weeks or 3 months. 34 out of 136 women (25%) who reported frequency of SM, removed at least once a day.
Handsearch	Meriwether et al, 2015b	Sexual function and pessary management among women who are using a pessary for pelvic floor	127 women presenting for a pessary fitting	Two tertiary care centres in USA	Secondary analysis of a randomized trial of new pessary users	Ring pessary, ring with support, ring with knob, incontinence dish, incontinence ring, Shaatz and short-stem Gellhorn	Not reported	Mean 56 years	3 months	Most common reason for r/o pessary, partner could feel during sex (20%) No women reported vaginal discharge or odour as reason for r/o pessary prior to sex. Daily pessary removal association with being sexually

For peer review only

		dysfunction								active at 3 months but did not improve sexual function or body image scores. For non-sexually active women, daily pessary removal associated with increased scores on partner related domain.
Handsearch	Tam et al, 2019	The effect of time interval of vaginal ring pessary replacement for pelvic organ prolapse on complication and patient satisfaction a randomised	60 women with stage 1-4 POP who opted for pessary management	Tertiary urogynecology center in Hong Kong	Double-blinded, randomized controlled trial	PVC ring pessary	88% stage 1-2	Mean 69 years	6 month	Self care of ring pessary performed by 2 out of 60 women. Many women refused to learn self-care.

For peer review only

		controlled trial								
Handsearch	Fregosi et al, 2018	Changes in the Vaginal Microenvironment as Related to Frequency of Pessary Removal	137 new pessary users attending pessary clinic	2 tertiary care centres in USA	Secondary analysis of RCT	Ring pessary, ring with support, ring with knob, incontinence dish, incontinence ring, Shaatz and short-stem Gellhorn	Not reported	Mean 59 years	3 month	34 (25%) removed the pessary daily, 54 (39%) at least weekly, and 49 (36%) less often than once weekly. Women who removed the pessary less often than weekly were older, using more hormone therapy, and more likely to have BV at baseline. Women who removed their pessaries less often than once weekly had an increased prevalence of anaerobes at 3 months, but no difference in vaginal

For peer review only

										symptoms or pessary satisfaction.
Handsearch	Clemons et al, 2004a	Patient characteristics associated with continued pessary use versus surgery after one year	67 women satisfied with pessary at 2 months for stage 2 or more POP	Urogynaecology clinic in USA	Prospective observational study	Ring and gell-horn	Median stage 3	Mean 72 years	12 months	Ability to change pessary not associated with continued pessary use or decision to opt for surgical management. SM women FU 6-12 monthly, otherwise 2-3 monthly.
Handsearch	Clemons et al, 2004b	Patient satisfaction and changes in prolapse and urinary symptoms in women who were fitted successfully with a pessary for pelvic	73 women with stage 2 or more prolapse successfully fitted with a pessary	Urogynaecology clinic in USA	Prospective observational study	Ring and gell-horn	Median stage 3	Mean 71 years	2 months	21 women (29%) were taught to insert and remove their pessary. All of the women taught to SM used ring pessaries because gell-horn are too difficult to SM.

For peer review only

		organ prolapse								
Handsearch	Kuhn et al, 2008	Sexual organ function in patients with symptomatic prolapse: are pessaries helpful?	73 women with stage 2 or more prolapse	Tertiary referral centre in Switzerland	Prospective observational study	Cube pessary	Not reported	Median 70 years	12 months	(Cube pessary) Patients received advice to remove the pessary as required for sexual intercourse
Handsearch	Bugge et al, 2013	Vaginal pessaries for pelvic organ prolapse and urinary incontinence: a multiprofessional survey of practice	678 members of RCOG, the Association for Continence Advice and the Association of Chartered Physiotherapists in Women's Health in the UK	Members of RCOG the Association for Continence Advice and the Association of Chartered Physiotherapists in Women'	Electronic questionnaire	Not applicable	Not applicable	Not applicable	Not applicable	17.7% (n=84) UK HCP recommend self-care following fitting. 4.1% (n=21) report patient responsible for pessary aftercare at organisation

				s Health in the UK.						
Handsearch	Thys et al, 2020	Effect of pessary cleaning and optimal time interval for follow-up a prospective cohort study	163 women with stage 2 or more primary prolapse fitted with pessary	Outpatient clinic at 3 teaching hospitals. The Netherlands.	Prospective cohort study	Silicone ring with or without support	Median stage 2	Mean 65 years	12 month follow-up	SM performed by 45.2% of women after one year. Patients who performed SM were younger, more frequently diagnosed with atrophy, reported less concomitant SUI and were more often sexually active. No difference between SM and non SM women in vaginal pain, discharge or irritation. No evidence for need to check asymptomatic patients performing SM with a silicone ring. SM more often sexually

										active including vaginal penetration.
Handsearch	Hanson et al, 2006	Vaginal pessaries in managing women with pelvic organ prolapse and urinary incontinence: patient characteristics and factors contributing to success	1216 women referred to nurse led pessary clinic	Nurse led pessary clinic in Canada	Retrospective notes review	Ring, ring with support, Gellhorn, incontinence dish, incontinence ring and the shaatz pessary	Not reported	Median 63 years	3 month follow-up	All patients were encouraged to learn to remove and reinsert the pessary weekly in order to clean the pessary and maintain their independence. Many patients were able to remove and clean their own pessary. Gell-horn and cube pessaries are the most difficult to remove, however a number of women were able to remove their gell-horn.
Handsearch	Abdool et al, 2011	Prospective evaluation of outcome of vaginal pessaries	554 women with symptomatic prolapse treated with either	Specialist urogynaecology unit UK	Prospective questionnaire study	Ring pessary, gellhorn pessary, cube, pessary and donut pessary.	Not reported	Mean 66 years	12 months	Many women preferred not to handle the pessary therefore a ring pessary was inserted for all

For peer review only

		versus surgery in women with symptomatic pelvic organ prolapse	surgery or pessary							sexually active women to avoid this.
Handsearch	Bai et al, 2005	Survey of the characteristics and satisfaction degree of the patients using a pessary	104 women fitted with a pessary and available for FU	Urogynaecology clinic South Korea	Retrospective review of notes and patient completed questionnaire	Ring pessary	Median 3	Mean 70 years	Not reported	The ring pessary is easy to wear and remove and effective. While the donut pessary is hard for a patient to remove by herself because the pessary does not have a notch. 19.1% of women removed their pessary (80% due to it slipping or discomfort)
Handsearch	Clemons et al, 2004c	Risk factors associated with an unsuccessful pessary fitting	100 consecutive women with symptomatic POP fitted with a pessary	Urogynaecology clinic in USA	Prospective observational study	Ring pessary and gell-horn pessary	Median 3	Mean 70 years	2 week	28 women (38%) were taught how to remove, clean and reinsert their pessary (ring or gell-horn pessary).

		trial in women with pelvic organ prolapse								
Handsearch	Sulak et al, 1993	Vaginal pessaries and their use in pelvic relaxation	101 women fitted with a pessary who returned for follow-up	Gynaecology clinic in USA	Retrospective review	Gell-horn pessary used for most participants, other pessary types used not reported	Not reported	Not reported	Not reported	5 of the 50 pessary users (10%) mentioned occasional difficulty with removal of the pessary (gell-horn). Another 5 had to have the pessary removed periodically by a nurse. For gell-horn, all patients (n=96) were advised to remove once or twice a week and leave it out overnight n=35 (70% removed the pessary at least once a week. The remainder removed it at least once every 6 weeks apart

For peer review only

										from one woman who had it in place for 6/12 without issue
Handsearch	*Ramsay et al, 2011	Long-term outcomes of pessary use in women with pelvic organ prolapse	429 women who underwent a pessary trial	Urogynaecology clinic in Hospital in Canada	Retrospective case notes review	Not reported	Not reported	Mean 71 years	Median 35 months	Pessary SM associated with prolonged pessary use. 66% of women were able to SM.
Handsearch	Brown et al, 2016	Defining patient's knowledge and perceptions of vaginal pessaries for prolapse and incontinence	254 women urogynaecology attendees	Urogynaecology clinic at an academic medical centre in the USA	Convenience sample questionnaire study	Not applicable- new patients to urogynaecology services	Not reported	Mean 57 years	No follow-up, data collection amongst new patients to service	Few women indicted concerns about pessary management for example that they would not be able to remove the pessary independently (n=20 16%).

For peer review only

Handsearch	Wu et al, 1997	A simplified protocol for pessary management	110 women receiving pessary management	Clinic in Canada	Prospective modified pessary care protocol followed and long term data collected at follow-up appointments	Ring with support, ring and cube	Unable to determine as reported as combined score for grade of prolapse in each compartment	Mean 65 years	Follow-up data collected up to 54 months	Ring pessary first choice as similar size and shape to diaphragm which patients find easy to insert and remove Most patients chose not to SM their pessary and return for regular office visits for pessary changes
Handsearch	Sarma et al, 2009	Long-term vaginal ring pessary use: discontinuation rates and adverse events	273 women fitted with a ring pessary in a tertiary urogynaecology clinic	Clinic in Australia	Retrospective notes review	Portex ring pessary or Introl bladder neck support device	Not reported	Mean 68 years	Median 7 years	All women who had reasonable manual dexterity were offered the option of self-management. Self-managing women were then seen 12 monthly.
Handsearch	Cundiff et al, 2000	A survey of pessary users by members of the American Urogynae	359 members of AUGS	AUGS members	Questionnaire study	Not applicable	Not applicable	Not applicable	Not applicable	53% (190) doctors reported teaching all patients to change their own pessary. 45% (162) reserve self-

For peer review only

		cology Society								management for a subset of women using support pessaries

For peer review only
